# Comparative study of the structural and optoelectronic properties of covalently bonded T-graphene-like bilayer nanoflakes: an *ab initio* investigation

**DOI:** 10.1039/d6ra04653c

**Published:** 2026-08-11

**Authors:** Abu Talha, Mohsina Faria Mou, Provati Rahman, Abdullah Al Roman, Debashis Roy, Mohammad Tanvir Ahmed

**Affiliations:** a Condensed Matter Physics (CMP) Lab, Department of Physics, Jashore University of Science and Technology Jashore 7408 Bangladesh debashisroy41ju@gmail.com mt.ahmed01@just.edu.bd

## Abstract

The exploration of novel two-dimensional (2D) materials with tunable properties is critical for advancing next-generation nano-electronics. In this study, we employ dispersion-corrected B3LYP-D3 *ab initio* calculations with HSEH1PBE single-point refinement to investigate the structural, electronic, and optical properties of novel tetragonal carbon-based molecular bilayers (MBLs), pristine TG, TBC_2_N, and heterostructures TG/TBN and TG/TBC_2_N. Geometry optimizations and frequency analysis confirm that all MBLs are dynamically and energetically stable. FMO analysis shows HOMOs localized on carbon-rich domains and LUMOs on B/N regions, indicating potential for directional charge transfer. Electronic property analyses demonstrate that all systems are semiconductors. The electronic energy gaps are highly tunable, ranging from 0.323 eV to 1.848 eV. MBL-TBC_2_N exhibits the highest reactivity, as shown by its low global hardness. UV-vis analysis confirms strong absorption in the UV and visible regions, with heteroatom doping and stacking inducing a significant redshift that broadens absorption. A critical finding from QTAIM analysis reveals the strong covalent bonds complemented by π–π* stacking and Reduced Density Gradient (RDG). These results highlight that these covalently-bonded MBLs are robust materials with highly tunable properties, possibly making them promising candidates for applications in sensing, catalysis, and nano-optoelectronics.

## Abstract 2

The exploration of novel two-dimensional (2D) materials with tunable properties is critical for advancing next-generation nano-electronics.

## Graphical abstract

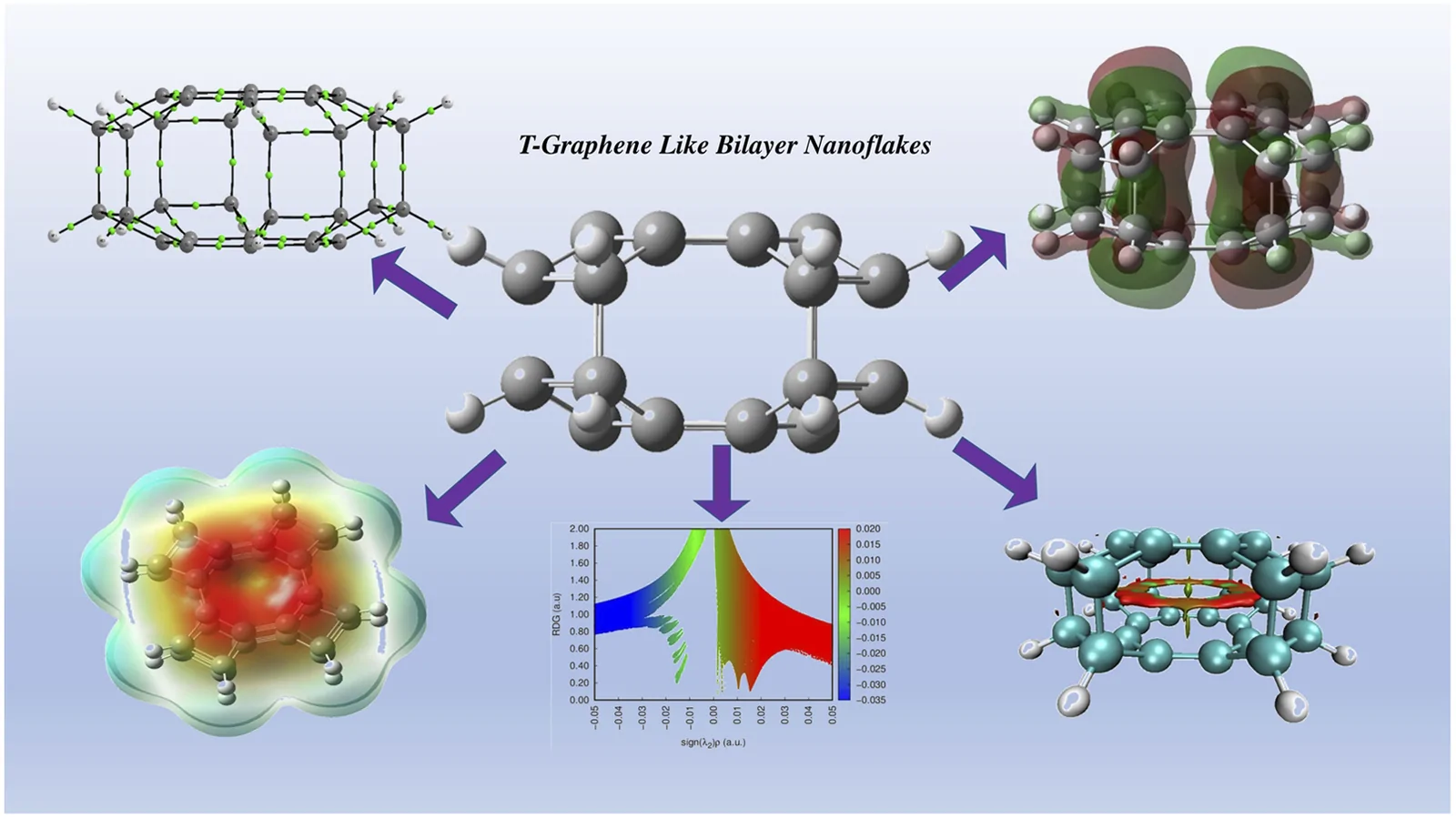

## 1 Introduction

Two-dimensional (2D) nanosheets constitute a recently developed class of nanomaterials characterized by atomic-scale thicknesses and large lateral dimensions (exceeding 100 nm), resulting in a high surface-to-volume ratio and conferring exceptional electronic, optical, and mechanical properties due to quantum confinement effects.^1–4^ Since the isolation of graphene, research has focused on developing new 2D systems with diverse lattice symmetries and chemical compositions to expand functional applications beyond traditional graphene-based systems.^5,6^ These materials exhibit tunable electronic and optical characteristics that may be relevant for future nano-electronic and optoelectronic applications.^7–9^

Graphene, consisting of sp^2^-hybridized carbon atoms arranged in a hexagonal lattice, exhibits ultrahigh carrier mobility, exceptional mechanical strength, and excellent thermal conductivity.^10,11^ However, its lack of an intrinsic energy gap limits its application in logic and switching devices, motivating the search for alternative carbon-based 2D allotropes. Notably, graphyne, graphdiyne, tetragonal graphene (TG), and other non-hexagonal carbon networks offer distinct bonding patterns and lattice symmetries that can induce an energy gap, anisotropic charge transport, and enhanced chemical reactivity.^12–15^ Theoretical studies suggest that these non-hexagonal carbon allotropes may outperform graphene in sensing and optoelectronic applications due to their improved tunability and higher surface activity.^12^ In addition, hexagonal boron nitride (h-BN) and boron–carbon–nitride (BCN) 2D materials have emerged as important functional systems. h-BN, a wide-band-gap insulator with excellent chemical and thermal stability, serves as a key dielectric in van der Waals heterostructures.^16,17^ Graphene and hexagonal boron nitride (h-BN) are two-dimensional materials with a low lattice mismatch (1.6–1.7%) and in-plane lattice constants of 2.46 Å and 2.51 Å, respectively; graphene is a zero-band-gap semiconductor, whereas h-BN is a wide-band-gap semiconductor. Both materials exhibit excellent chemical and mechanical stability along with outstanding electrical, optical, and thermal properties, and the carbon atoms in graphene are covalently bonded.^1,18^ The recent integration of h-BN with graphene has enabled the effective modulation of the h-BN energy gap, primarily through the incorporation of carbon atoms into the h-BN lattice and the subsequent formation of complex interfacial BxCyNz layers.^19^ Boron nitride (BN) nanomaterials, including BN clusters, BN nanoparticles, BN nanocapsules, and BN nanotubes, have been extensively studied. These materials are expected to be useful for gas storage applications, insulating lubricants, heat-resistant semiconductors, and electronic devices.^20,21^ BCN materials allow continuous tuning of electronic properties by adjusting the B : C : N ratio, enabling band-gap engineering, charge redistribution, and enhanced optical absorption, which have been utilized in optoelectronic and sensing applications.^22,23^

Beyond monolayers, bilayer and few-layer 2D materials provide additional flexibility through interlayer coupling, stacking sequence, and symmetry effects. For example, bilayer graphene can exhibit a tunable energy gap under an external electric field, a property absent in the monolayer, highlighting the significant influence of interlayer interactions.^2,24^ In graphene, monolayers exhibit Dirac-like linear band dispersion, while bilayers and few layers show tunable energy gaps under electric fields or *via* stacking arrangements (*e.g.*, AB, AA, twisted), opening new physics such as correlated states and potential superconductivity.^25^ In h-BN, increasing layer number enhances mechanical robustness and dielectric screening, while preserving its insulating nature, making it useful in layered devices where insulation and interface quality are critical.^26^ Similarly, bilayer BN and BCN exhibit modified dielectric screening, altered optical transitions, and enhanced structural stability due to stacking interactions.^24,27,28^ However, strong interlayer coupling can sometimes reduce carrier mobility or surface reactivity, indicating that the performance of bilayer systems depends strongly on lattice symmetry and bonding.^29^

While most bilayer studies have concentrated on hexagonal lattices, low-symmetry 2D materials, particularly tetragonal structures, remain underexplored. Tetragonal 2D materials, characterized by square or rectangular atomic arrangements, exhibit reduced crystallographic symmetry, leading to anisotropic electronic dispersion, optical behavior, and chemical bonding.^30^ This anisotropy enables direction-dependent charge transport and unconventional optical responses, making these materials promising for advanced electronic, optoelectronic, and sensing devices. First-principles studies show that tetragonal monolayers can possess stable semiconducting behavior, enhanced chemical activity, and tunable electronic properties compared to hexagonal counterparts.^7^

When extended to bilayer configurations, tetragonal systems are expected to display distinct interlayer interactions, including pronounced band-structure reconstruction, enhanced charge transfer, and tunable carrier masses. Additionally, bilayer formation can improve mechanical stability and suppress out-of-plane instabilities, increasing practical applicability.^23^

Despite these promising characteristics, systematic investigations of tetragonal bilayer 2D materials remain limited, with most studies focusing on monolayer properties and leaving the stability of bilayers, interlayer coupling effects, and device-relevant performance largely unexplored. This gap motivates further study to establish clear structure–property relationships and assess the technological potential of tetragonal bilayer systems. It should be noted that the present study is based on finite molecular bilayer nanoflake (MBL) models, which are used as representative clusters to mimic local properties of extended 2D systems. Therefore, all conclusions are limited to nanoscale behavior and should not be interpreted as direct properties of infinite periodic monolayers or bulk 2D sheets.

In this work, we present a comprehensive density functional theory (DFT) study on tetragonal carbon-based bilayers, including pristine TG, TBC_2_N, and heterostructures TG/TBN and TG/TBC_2_N. To clarify interlayer interactions, Quantum Theory of Atoms in Molecules (QTAIM) and reduced density gradient (RDG) analyses are employed. To our knowledge, this is the first systematic theoretical investigation of these tetragonal bilayer systems.

## 2 Computational methodology

The molecular bilayer (MBL) systems were investigated using density functional theory (DFT) calculations implemented in the Gaussian 16 package.^31^ Geometry optimizations were carried out using the dispersion-corrected B3LYP-D3 hybrid functional together with the 6-311G(d, p) basis set.^32–34^ Additional calculations using the Lanl2DZ basis set are included in the SI to further support the computational methodology and the reliability of the reported results. Frequency calculations were subsequently performed at the same level of theory to confirm the absence of imaginary frequencies and to obtain the corresponding thermodynamic parameters, ensuring that the optimized structures correspond to true minima on the potential energy surface. To achieve more reliable predictions of the electronic properties, particularly the highest occupied molecular orbital (HOMO) and the lowest unoccupied molecular orbital (LUMO) gaps, single-point energy calculations were further refined using the HSEH1PBE hybrid functional with the same basis set.^35^

The cohesive energy (*E*_Coh_) of MBLs was determined according to the following relations:^7^1
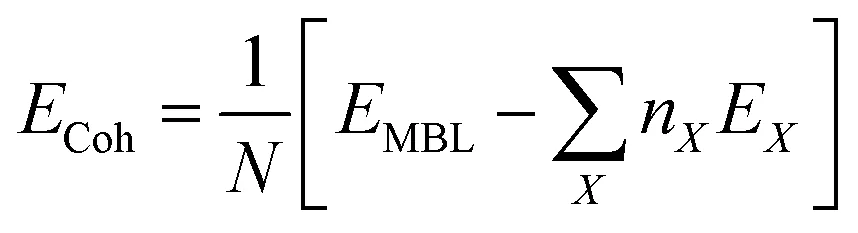
*E*_MBL_, *N*, *n*_*X*_, and *E*_*X*_ denote the total energies of the MBLs, is the total number of atoms in the MBL systems, *n*_*X*_ represents the number of atoms of type *X* (C, N, B, and H), and is the *E*_*X*_ energy of the isolated atom of type *X*.

The binding energy (*E*_BE_) for the MBLs was computed according to the following relation:^36^2
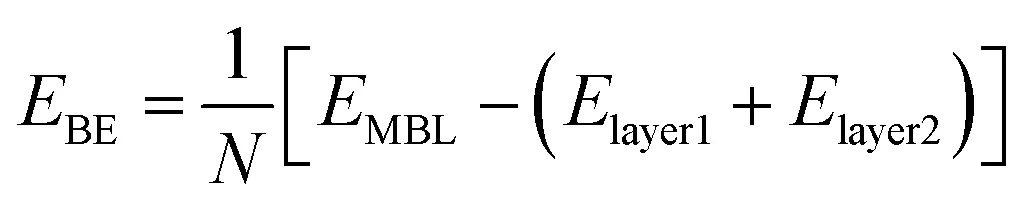
where, *E*_BE_ is the binding energy per atom, *E*_bilayer_ (*E*_MBL_) is the total energy of the optimized bilayer, *E*_layer1_ and *E*_layer2_ are the total energies of the isolated first and second monolayers, respectively, and *N* is the total number of atoms in the bilayer.

The electronic and chemical reactivity descriptors were derived from the frontier molecular orbitals, *i.e.*, the HOMO and the LUMO. The following relationships were employed:^37^

3 *E*_g_ = *E*_LU_ − *E*_HO_

4
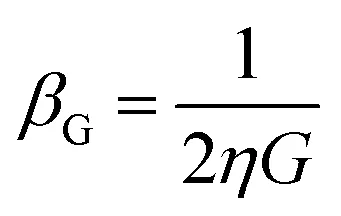
5
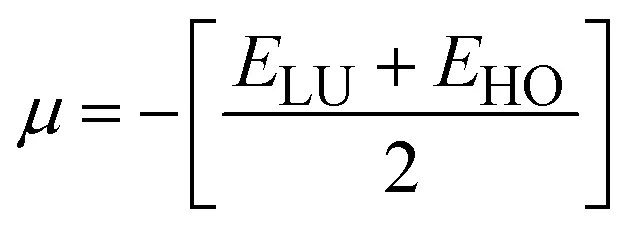
6
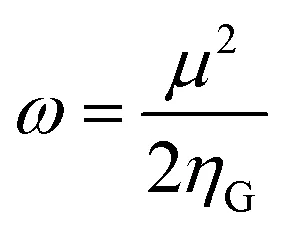
where *E*_*g*_, *E*_LU_, *E*_HO_ and *E*_f_ are the energy gap, LUMO energy, HOMO energy, and Fermi energy, respectively. The descriptors *η*_G_, *β*_G_, *µ*, and *ω* represent the global hardness, global softness, chemical potential, and electrophilicity index, respectively.

Finally, the nature of interlayer interactions and noncovalent electronic features was explored using the quantum theory of atoms in molecules (QTAIM) and reduced density gradient (RDG) analyses implemented in the Multiwfn program.^34,38^

## 3 Results and discussion

### 3.1 Geometrical stability of bilayer-like structures

**Fig. 1:**
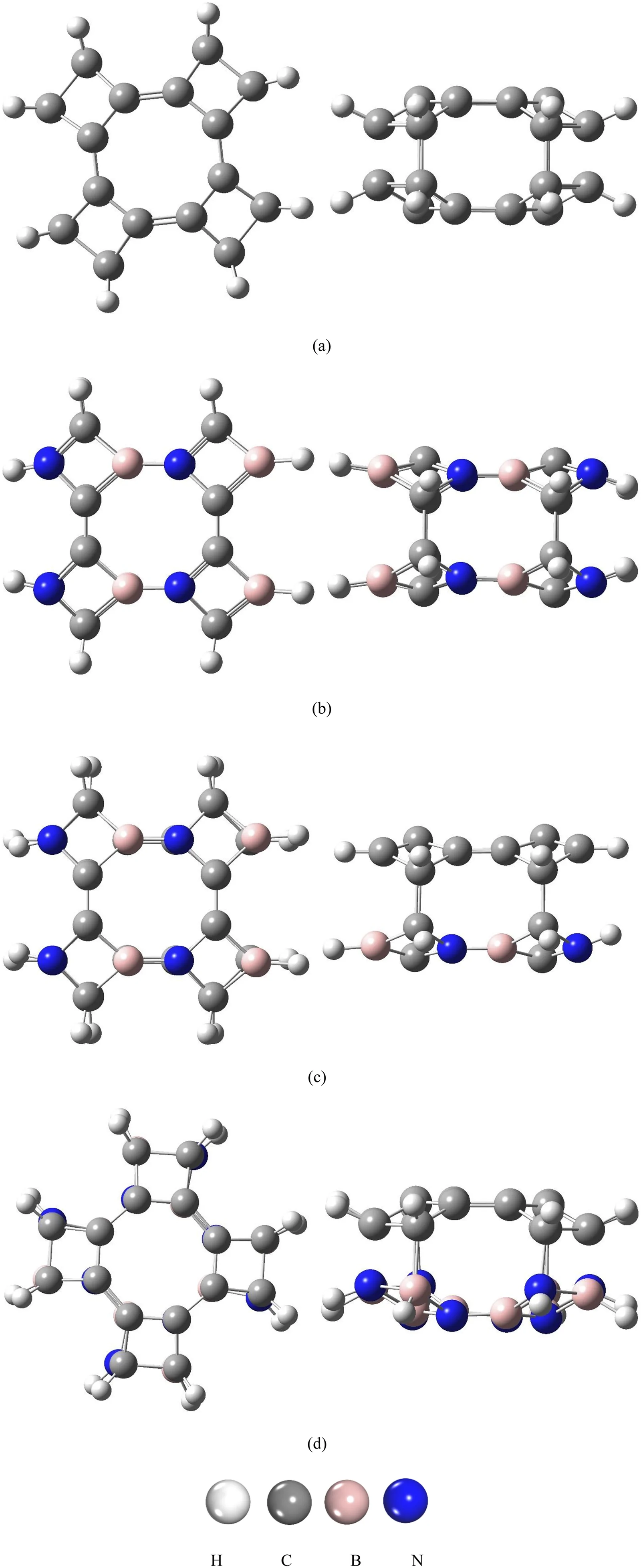
Optimized geometries of the MBL systems after full structural relaxation: (a) TG, (b) TBC_2_N, (c) TG/TBC_2_N, (d) TG/TBN.

The geometry optimization results confirm that all Molecular Bilayer (MBL) systems, twin MBL-TG, MBL-TBC_2_N, and their heterostructures MBL-TG/TBN and MBL-TG/TBC_2_N, are structurally stable (Fig. 1). Moreover, the cohesive energies per atom of the MBL systems with the 6-311G(d,p) basis set are −6.5354 eV, −6.2177 eV, −6.1436 eV, and −5.8872 eV for TG, TG/TBC_2_N, TG/TBN, and TBC_2_N, respectively, and the binding energies per atom of the MBL systems are −0.4652 eV, −0.4178, −0.363 eV, −0.3575 eV for TG, TG/TBC_2_N, TG/TBN, and TBC_2_N, respectively. The significantly negative values further confirm that all MBL systems are energetically stable.^39^

All geometries converged without lattice collapse, and the bond lengths in both tetragonal (4-membered) and octagonal (8-membered) units remained within the typical covalent range in both L1 and L2 layers. The shared bonds between octagonal and tetragonal rings located at the junctions of the tetragonal and octagonal rings play a crucial role in maintaining the stability of the structures, and no significant distortions are observed after relaxation. In MBL-TG, within both L1 and L2 layers, the in-plane C–C bonds remain uniform at ∼1.55 Å, consistent with earlier theoretical reports on tetragonal graphene.^40–42^ In MBL-TBC_2_N, substitution with B and N atoms modifies the bonding environment: C–B bonds elongate to ∼1.59 Å, while C–N bonds shorten to ∼1.49 Å, reflecting the combined effects of atomic size and electronegativity differences (Table 1).

**Table 1:** Bond lengths between C–C, C–B, C–N, and B–N atoms in tetragonal, octagonal, and shared structures of the MBL framework in two layers (L1 and L2)

Structure	Layer	Bond length									
		Tetragonal				Octagonal			Common bonds		
		C–C	C–B	C–N	B–N	C–C	B–N	C–C	C–B	C–N	B–N
TG	L1	1.53	—	—	—	1.46	—	1.36	—	—	—
	L2	1.53	—	—	—	1.46	—	1.36	—	—	—
TBC_2_N	L1	—	1.58	1.47	—	1.36	1.41	—	1.54	1.45	—
	L2	—	1.58	1.47	—	1.36	1.41	—	1.54	1.45	—
TG/TBC_2_N	L1	1.48	—	—	—	1.33	—	1.45	—	—	—
	L2	—	1.58	1.47	1.40	1.39	1.41	—	1.53	1.44	—
TG/TBN	L1	1.53	—	—	—	1.43	—	1.41	—	—	—
	L2	—	—	—	1.61	—	1.41	—	—	—	1.44

In the hybrid bilayers, intermediate features are observed. For example, MBL-TG/TBN exhibits B–N bonds of ∼1.61 Å in the tetragonal unit and ∼1.41 Å in the octagonal unit of the L2 layer, accompanied by only small changes in the C–C bonds of the L1 layer compared to MBL-TG. In contrast, MBL-TG/TBC_2_N contains a more diverse set of linkages: in the L1 layer, C–C bonds range between 1.33 and 1.48 Å, while in the L2 layer, C–B bonds appear between 1.54 and 1.58 Å, C–N bonds between 1.44 and 1.47 Å, and B–N bonds around 1.40 Å. The persistence of these bond lengths within stable covalent ranges demonstrates that the coexistence of tetragonal and octagonal units enhances the robustness of the molecular bilayer structures. The coexistence of different bond types also reflects the integrated structural features of carbon-rich and BN-rich domains, confirming that the hybrid bilayers achieve stability through a diverse set of interlayer interactions.

As discussed in the QTAIM and RDG analysis section, the interlayer (L1–L2) bonding is not governed by weak van der Waals forces but is instead stabilized through a combination of covalent linkages and π–π* interactions, which collectively enhance the structural stability of the molecular bilayer structures. The overall absence of significant structural deformation or bond instability further confirms that all bilayer systems achieve geometric stability.

### 3.2 Infrared (IR) activity of bilayer structures

The infrared vibrational analysis validates the dynamical stability of all Molecular Bilayer (MBL) systems, as no imaginary frequencies were detected, confirming that the optimized structures represent true minima on the potential energy surface cite. Each bilayer shows distinct vibrational signatures that are consistent with reported experimental and theoretical ranges. In addition, sp^2^ C–H stretching VFs are observed at 3080.14–3104.16 cm^−1^, in agreement with reported values for γ-SiC nanoflakes (2999–3021 cm^−1^) and other sp^2^ carbon systems (∼3100–3312 cm^−1^) (Table 2).

**Table 2:** Calculated molecular vibrational frequencies (cm^−1^) following structural optimization

MBL structures	Vibration type	Frequencies of MBL structures (cm^−1^)		
		Present study	Referenced structure	Literature
TG	sp^2^ C–C stretching	1595.27–1610.97	Amorphous carbon nitrides	1500–1600 (ref. 40)
	sp^2^ C–H stretching	3080.14–3104.16	γ-SiC nanoflake	3020.9 (ref. 34)
	Inter-layer stretching	288.32(in/out) 331.32(L1-L2)	—	—
TBC_2_N	sp^2^ C–C stretching	1603.55–1725.45	Amorphous carbon nitrides	1500–1600 (ref. 40)
	B–N stretching	1408.38–1516.68	sp^2^ Bonded B–N (in hexagonal BN (pristine mono- and bilayers)	1350–1500 (ref. 41)
	B–H stretching	2691.68–2703.68	BH_3_–NH_3_ in the solid state	2592–2607 (ref. 42)
	N–H stretching	3471.34–3479.40	h-BN	∼3500–3300 (ref. 43)
	sp^2^ C–H stretching	3031.96–3071.39	γ-SiC nanoflake	3263, 3312 (ref. 34)
	Inter-layer stretching	588.35	—	—
TG/TBC_2_N	sp^2^ C–C stretching	1530.14–1752.36	Amorphous carbon nitrides	1500–1600 (ref. 40)
	B–N stretching	1429.53–1486.98	sp^2^-Bonded hexagonal boron nitride	1350–1500 (ref. 41)
	B–H stretching	2679.93–2685.36	h-BN, BH_3_–NH_3_ in the solid state	∼2500 (ref. 44), 2592–2607 (ref. 42)
	N–H stretching	3468.89–3476.97	h-BN	∼3300–3500 (ref. 43 and 44)
	sp^2^ C–H stretching	3042.49–3205.42	γ-SiC nanoflake	3312 (ref. 34)
	Inter-layer L1–L2 stretching	605.41	—	—
TG/TBN	sp^2^ C–C stretching/bending	1508.23–1599.79	Amorphous carbon nitrides	1500–1600 (ref. 40)
	B–N stretching	1354.73, (1443.24–1521.43)	sp^2^-Bonded hexagonal boron nitride	1350–1500 (ref. 41)
	B–H stretching	2539.37–2543.19	h-BN, BH_3_–NH3 in the solid state	∼2500 (ref. 44), 2592–2607 (ref. 42)
	N–H stretching	3559.79–3559.81	h-BN	∼3300–3500, (ref. 44) 3800 (ref. 43)
	sp^2^ C–H stretching	3085.68–3100.63	γ-SiC nanoflake	3263, 3312 (ref. 34)
	Inter-layer L1–L2 stretching	284.32	—	—

**Fig. 2:**
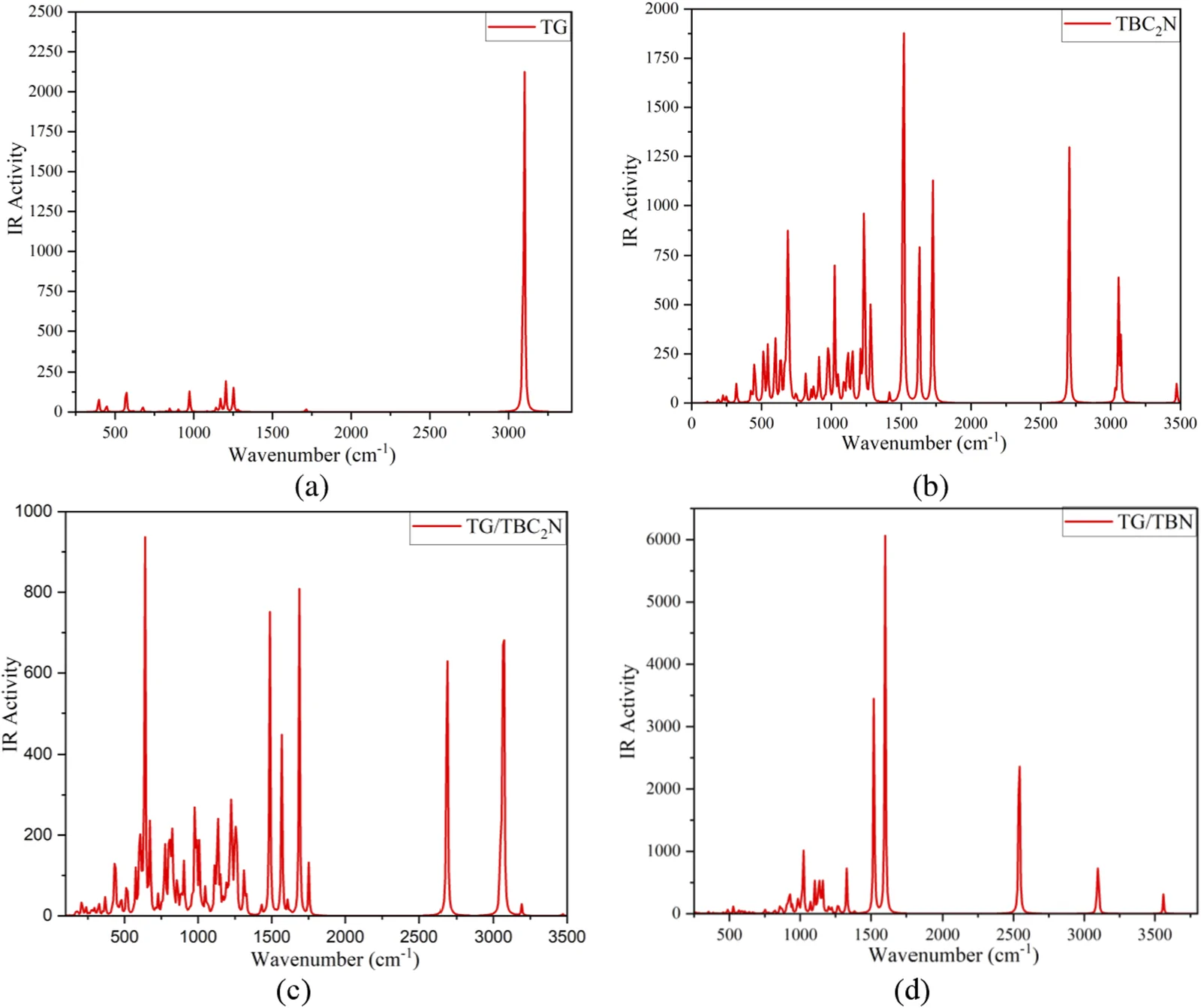
Infrared (IR) spectra of MBL systems: (a) TG, (b) TBC_2_N, (c) TG/TBC_2_N, (d) TG/TBN.

For all MBL systems, the IR spectra are mainly characterized by sp^2^ C–C stretching VF in the nearly 1508.23–1752.36 cm^−1^ region, which falls within the typical range reported for sp^2^ C–C frameworks in amorphous carbon nitrides close to (1500–1600 cm^−1^).^40^ In addition, for MBL-TG, C–H stretching bands appear between 3080.14 and 3104.16 cm^−1^, in agreement with earlier studies on γ-SiC Nanoflake.^34^ For MBL-TBC_2_N, new vibrational features emerge from the incorporation of B and N. The B–N stretching bands (1408.38–1516.68 cm^−1^) are consistent with sp^2^-bonded BN in hexagonal BN monolayers and bilayers (1350–1500 cm^−1^).^40^ The B–H stretching modes (2691.68–2703.68 cm^−1^) correspond well with experimental values for BH_3_–NH_3_ solids (2592–2607 cm^−1^), while the N–H stretching modes (3471.34–3479.40 cm^−1^) match literature values for h-BN (∼3300–3500 cm^−1^).^46–48^ The C–H stretching region (3031.96–3071.39 cm^−1^) also agrees with the reported range for γ-SiC nanoflakes and carbon nanostructures (2999–3312 cm^−1^).^37^ For MBL-TG/TBC_2_N and MBL-TG/TBN, nearly similar vibrational signatures are observed, with B–N, B–H, N–H, and C–H stretching VFs appearing in the same ranges as in MBL-TBC_2_N, confirming the combined structural characteristics of carbon- and BN-rich domains. Various inter-layer stretching vibrational frequencies (VFs) are observed for the MBL systems. For TG, the inter-layer stretching VFs are 288.32 cm^−1^ (in-plane/out-of-plane) and 331.32 cm^−1^ (between L1 and L2 layers). TBC_2_N exhibits an inter-layer stretching VF of 588.35 cm^−1^, while the TG/TBC_2_N heterostructure shows 605.41 cm^−1^. For TG/TBN, the inter-layer stretching VF is 284.32 cm^−1^ (Fig. 2).

Overall, the observed vibrational ranges are in close agreement with both experimental and theoretical values, validating the robustness of the bilayer frameworks. They also highlight the influence of B and N incorporation on the bonding environment. These IR-active modes not only confirm the stability of the optimized structures but also serve as reliable spectroscopic markers for distinguishing between different MBL systems. The calculated cohesive energies and vibrational analyses indicate that the proposed MBL nanoflakes are energetically favorable and dynamically stable at the optimized geometries. These results provide evidence for the local stability of the structures, although further investigations involving binding energies and finite-temperature simulations would be necessary to establish their complete thermodynamic stability.

### 3.3 Electronic properties

#### 3.3.1 Mulliken charge analysis

**Fig. 3:**
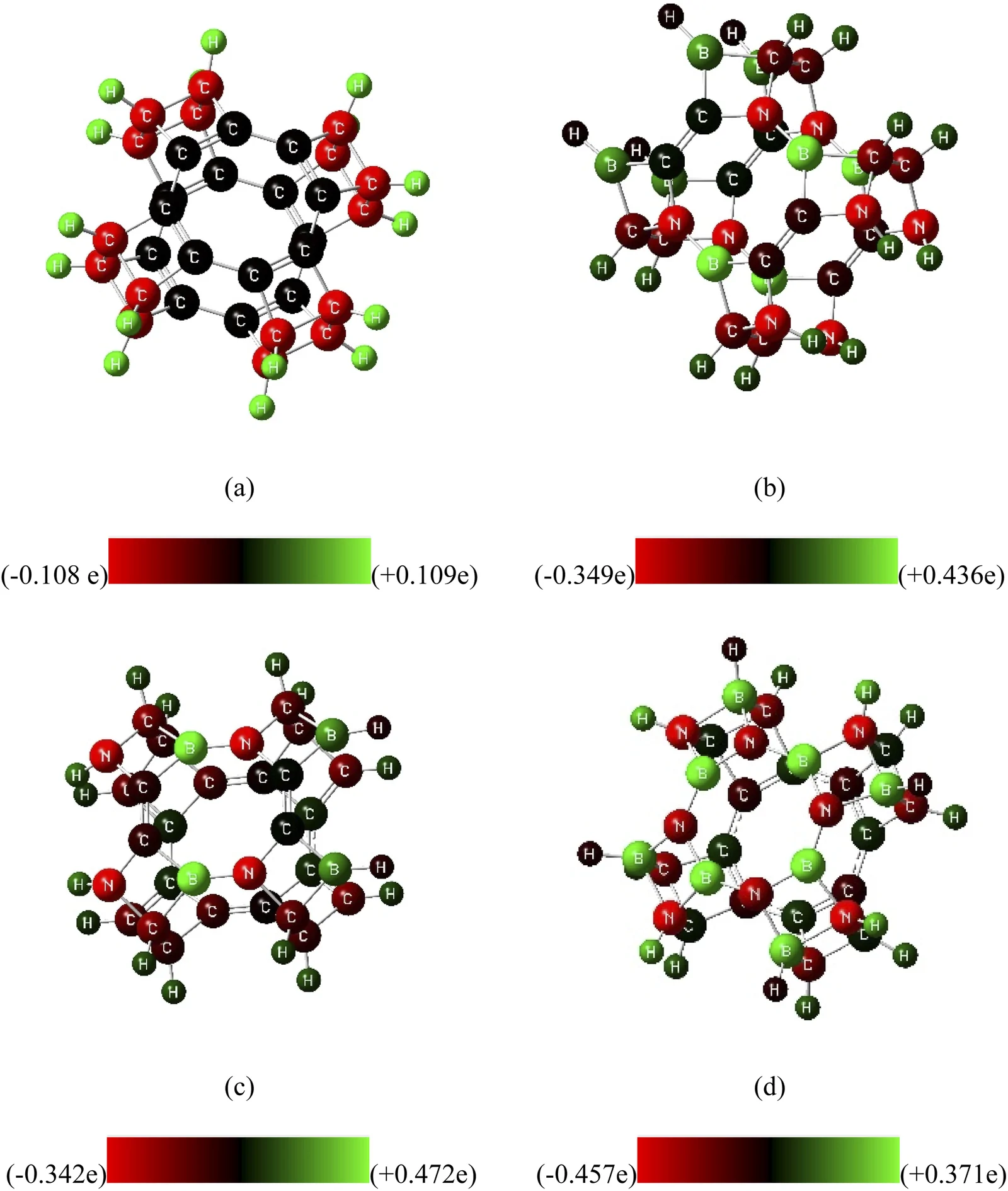
Distribution of Mulliken charges in the optimized MBL structures: (a) TG, (b) TBC_2_N, (c) TG/TBC_2_N, (d) TG/TBN.

Fig. 3 illustrates the Mulliken charge distribution for the MBL substrates, highlighting how variations in local electron density correlate with the electronegativities of the constituent atoms. Atoms with higher electronegativity tend to attract bonding electrons more strongly, resulting in a partial negative charge. Within the TG layer, carbon atoms located at the central or octagonal sites exhibit nearly neutral charges, as they are surrounded by identical atoms, leading to minimal net electron transfer. In contrast, carbon atoms at the sheet edges, bonded to hydrogen, show slight electron accumulation due to the higher electronegativity of carbon relative to hydrogen. Consequently, these edge carbon atoms appear as electron-rich regions (red) on the charge map, whereas hydrogen atoms appear electron-deficient (green).

A comparable trend is observed for TBC_2_N, where the highly electronegative nitrogen atoms at the edges draw electron density from neighboring boron, carbon, and hydrogen atoms, generating a pronounced negative potential around the nitrogen sites. In the TG/TBC_2_N and TG/TBN heterostructures, the TG sublayer remains largely neutral owing to the uniform carbon environment. Meanwhile, the adjacent TBC_2_N or TBN layers exhibit increased polarity, with edge nitrogen atoms dominating the electron distribution. These nitrogen sites are depicted as strongly electron-rich (red), whereas the surrounding carbon, boron, and hydrogen atoms, being less electronegative, display a gradient toward electron deficiency (green).

#### 3.3.2 HOMO and LUMO

**Fig. 4:**
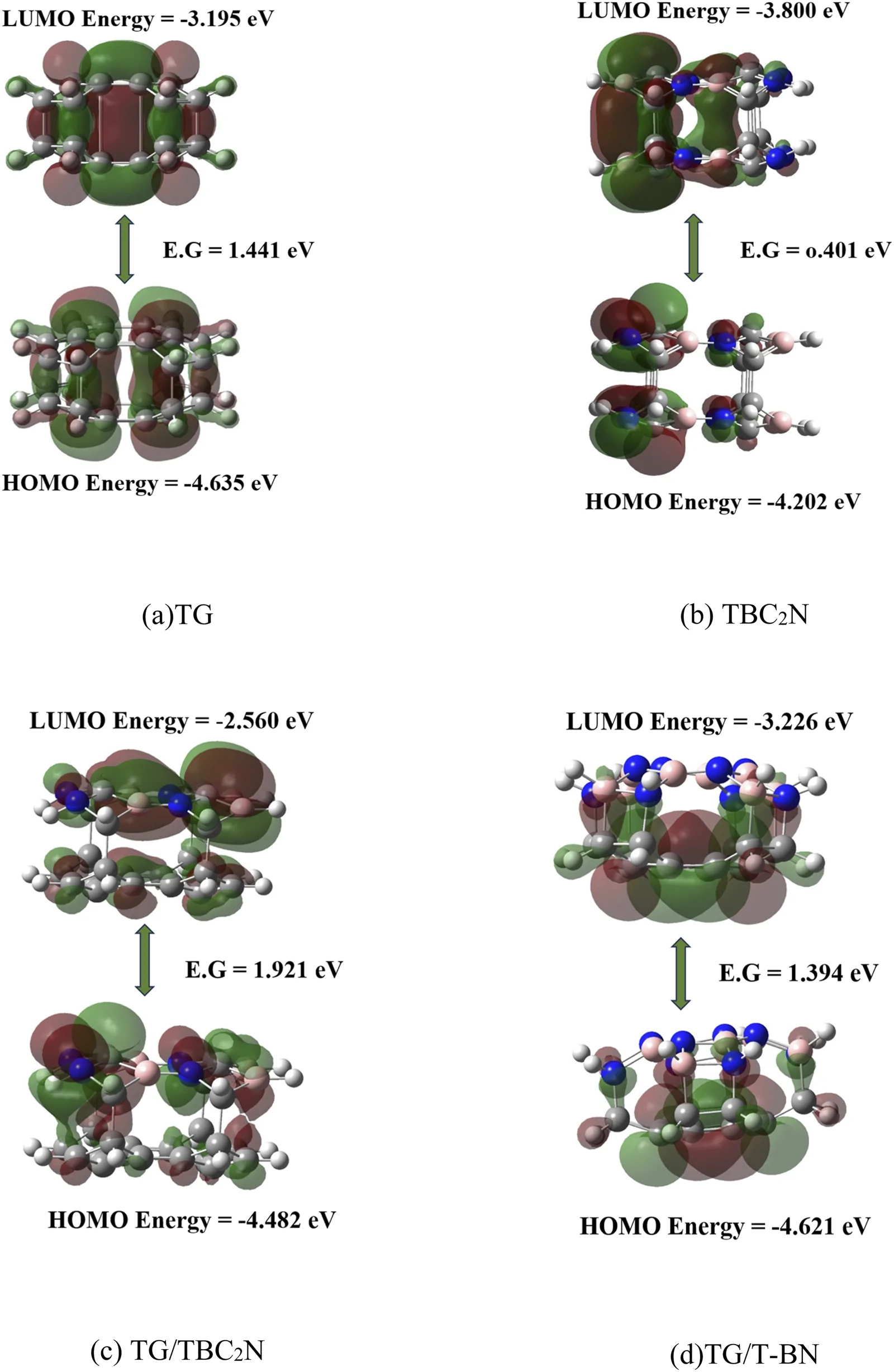
Molecular orbitals of the MBL structures: (a) TG, (b) TBC_2_N, (c) TG/TBC_2_N, (d) TG/TBN, generated with an isovalue 0.02.

The frontier molecular orbitals of the investigated bilayer systems provide essential insight into their electronic activity and intrinsic reactivity. In all structures, the highest occupied molecular orbital (HOMO) is primarily delocalized over the carbon-rich domains, whereas the lowest unoccupied molecular orbital (LUMO) shows enhanced localization near the heteroatomic (B/N-containing) regions (Fig. 4). The green and red colors represent opposite phases of the molecular orbitals and are used to visualize the spatial distribution and symmetry of the HOMO and LUMO wavefunctions. This orbital distribution highlights the role of B and N doping in tailoring charge-transport channels and polarization within the frameworks.

Pristine TG exhibits a moderate energy gap of 1.441 eV, indicative of semiconducting behavior. The incorporation of B and N significantly narrows the energy gap in TBC_2_N to 0.401 eV, reflecting improved charge mobility and higher polarizability as a consequence of symmetry breaking and heteroatom-induced electronic perturbation. The TG/TBN and TG/TBC_2_N heterostructures exhibit intermediate band gaps of 1.394 eV and 1.921 eV, respectively, indicating that hybrid stacking effectively tunes the electronic properties of the systems (Table 3). The energy gap of the TG bilayer structure is comparatively higher than that of the single-layer graphene (*G*), which is a gapless semimetal characterized by Dirac cones at the *K* point, resulting in a zero-energy gap,^45^ various monolayer *t*-graphene-based nanomaterials^39,46^ and also the graphene–graphene (G–G) bilayers, whose energy gap is typically near 0 eV.^47^ The energy gap of the entire structure is comparatively smaller than that of single-layer γ-graphyne (2.604 eV),^48^ pristine graphene (4.87 eV),^9^ penta-graphene (2.219 eV),^49^ boron carbon nitride nanosheet (2.046 eV),^4^ monolayer hexagonal boron nitride (h-BN) (4.96 eV),^50^ tetragonal boron nitride nanosheet (4.10 eV),^51^ and the BN bilayer (3.814).^20^ These energy gap variations imply tunability of electrochemical and sensing performance by heteroatom substitution and bilayer coupling.

**Table 3:** HOMO energy, LUMO energy, and energy gap of studied MBL structures

Structures	HOMO energy (eV)	LUMO energy (eV)	HOMO LUMO energy gap (eV)
TG	−4.635	−3.195	1.441
TBC_2_N	−4.202	−3.800	0.401
TG/TBN	−4.621	−3.226	1.394
TG/TBC_2_N	−4.482	−2.560	1.921

Overall, the orbital profiles reveal directional charge transfer capability, where electrons preferentially accumulate at nitrogen centers in the conduction state. This orbital pattern suggests favorable conditions for electron–acceptor interactions, relevant for sensing and catalytic applications.

#### 3.3.3 Electrostatic potential (ESP) map

**Fig. 5:**
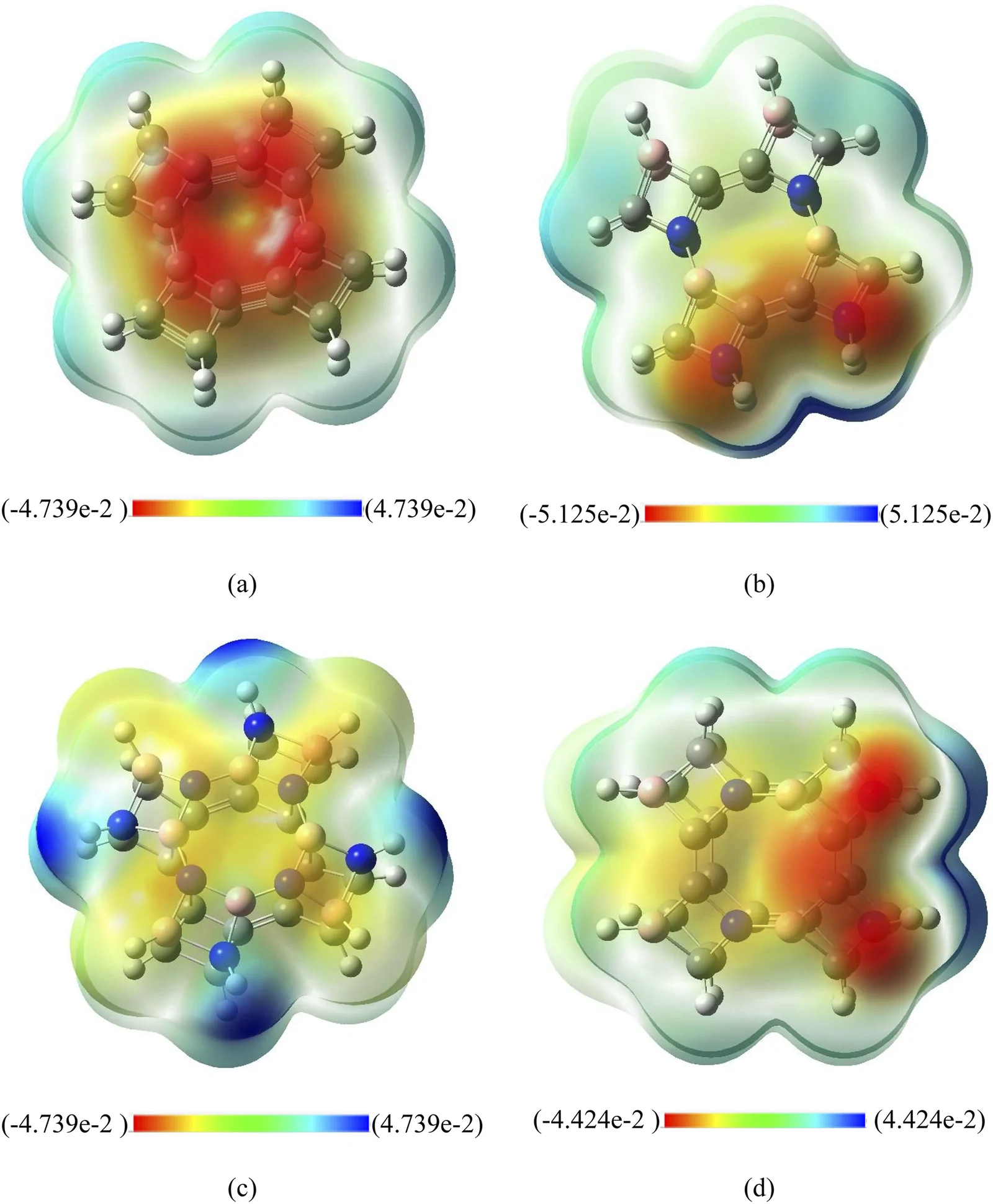
Electrostatic potential map of studied MBL structures: (a) TG, (b) TBC_2_N, (c) TG/TBN, (d) TG/TBC_2_N.

The electron density maps are characterized by two distinct regions: red zones correspond to electron-rich areas that are prone to electrophilic attack (EZ), whereas blue zones indicate electron-deficient regions susceptible to nucleophilic attack (NZ). In Fig. 5, the pristine MBLs display EZ and NZ predominantly at diagonally opposite regions of the surface. The electrostatic potential maps further verify the charge distribution across the bilayer frameworks. Strongly negative potential regions are observed around nitrogen sites, while boron-rich regions appear notably electron-deficient. Carbon-dominated domains exhibit a comparatively neutral potential landscape.

In the TG monolayer, the electron density is largely uniform, except for slight localization at the edge hydrogen atoms. In contrast, the TBC_2_N and TG/TBN heterostructures exhibit pronounced electronic polarization. The nitrogen-enriched edges generate electron-rich regions that can readily participate in electrophilic interactions. Within the mixed bilayer systems, the TG layer preserves its overall charge neutrality, while significant charge accumulation occurs in the adjacent B/N-containing layers. This behavior indicates the presence of asymmetric charge distributions resulting from both heteroatom doping and interlayer coupling effects.

These ESP characteristics are consistent with the strong interlayer interactions identified through QTAIM analysis and indicate the existence of regions with enhanced surface polarity and localized charge distribution. The simultaneous presence of electron-rich and electron-poor domains further highlights the potential of these systems for charge-transfer-driven sensing and catalytic applications.

#### 3.3.4 Density of states (DOS)

**Fig. 6:**
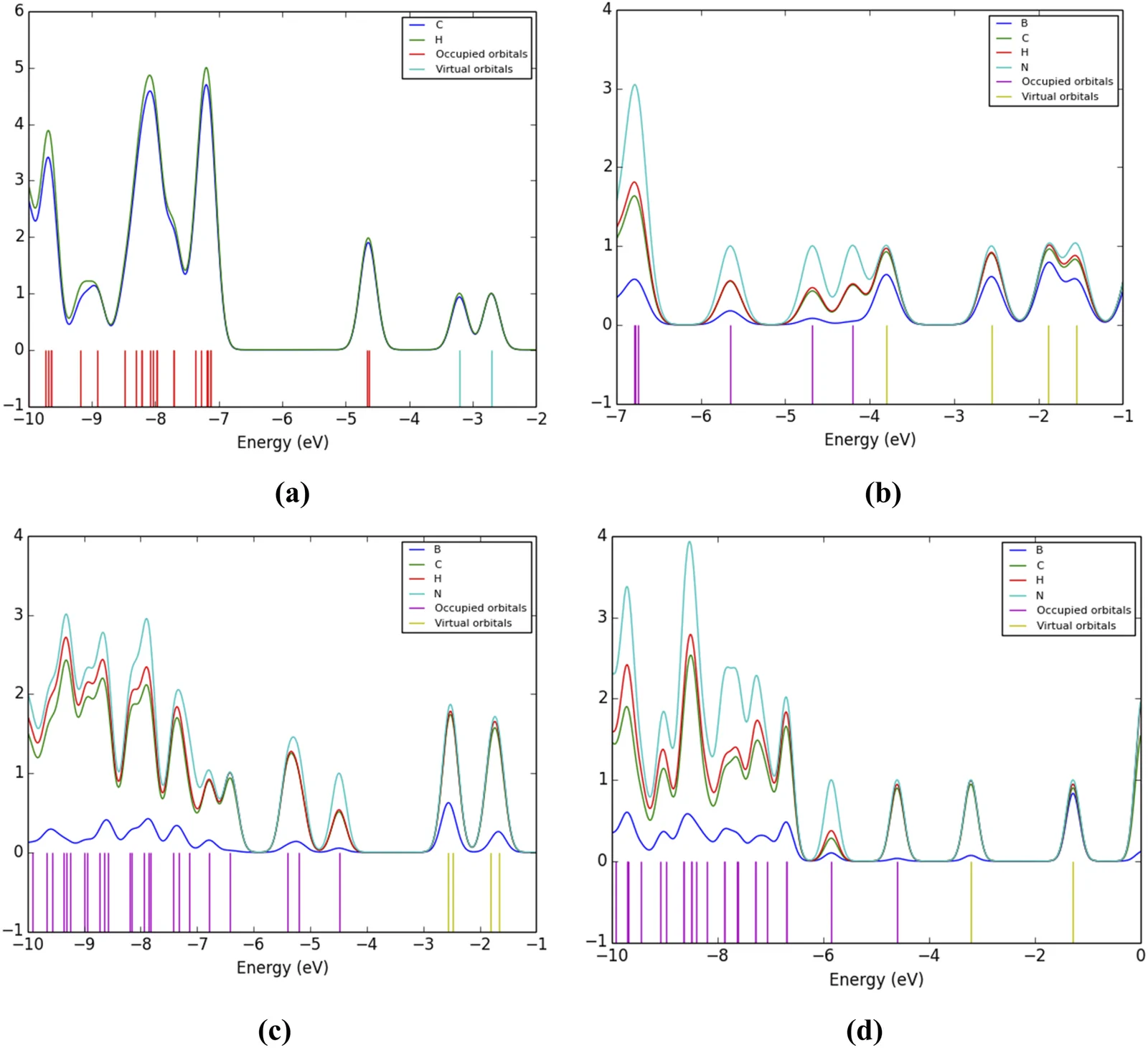
Projected density of states of studied MBL structures: (a) TG, (b) TBC_2_N, (c) TG/TBC_2_N, (d) TG/TBN.

The Projected Density of States (PDOS) spectra provide a deeper understanding of the orbital contributions around the Fermi level. The PDOS spectra were plotted by taking the Fermi level as the zero-energy reference. A Gaussian broadening scheme was employed to visualize the discrete molecular orbital contributions near the frontier levels. Since the present study considers finite molecular nanoflakes rather than periodic crystalline systems, the reported electronic gaps correspond to HOMO–LUMO gaps derived from molecular orbital energies rather than true periodic band structures. All MBL systems exhibit a distinct energy gap region with nearly zero density of states, confirming their semiconducting nature.^34^ In pristine TG, the states near the Fermi level are primarily derived from C-2p orbitals, whereas the introduction of boron and nitrogen in the doped and hybrid configurations adds new electronic contributions from their respective p-orbitals. For MBL-TBC_2_N, the noticeable reduction in energy gap and the higher state density near the frontier levels indicate enhanced orbital hybridization and charge delocalization, correlating with its greater electronic reactivity. The hybrid bilayers display redistributed PDOS patterns, signifying strong electronic coupling between the constituent layers. In particular, MBL-TG/TBC_2_N shows distinct, sharper peaks near the conduction band edge, reflecting strong orbital interactions between carbon and heteroatom domains (Fig. 6).

Overall, the subtle peak shifts and intensity variations observed across the MBL systems indicate that heteroatom substitution and bilayer formation effectively modulate the orbital landscape. Such electronic tuning enhances interlayer charge-transfer characteristics and strengthens electronic coupling between the neighboring layers. Consequently, these modifications are expected to improve electronic sensitivity and electrical response, highlighting the tunability of their electronic properties for potential low-dimensional electronic applications.

### 3.4 Global indices

The quantum chemical global reactivity descriptors, global hardness (*η*_G_), global softness (*β*_G_), chemical potential (*µ*), and electrophilicity index (*ω*), are essential for evaluating the chemical stability, reactivity, and interaction potential of nanomaterials. These indices were determined using frontier molecular orbital (FMO) energies and are summarized in Table 4.

**Table 4:** Global indices of the optimized geometries. Global hardness *η*_G_, global softness *β*_G_, chemical potential *µ*, the electrophilicity index of the structures *ω*

MBL structures	Global hardness, *η*_G_ (eV)	Global softness, *β*_G_ (eV^−1^)	Chemical potential, *µ* (eV)	Electrophilicity index of the structures, *ω* (eV)
TG	0.720	0.694	3.915	10.639
TBC_2_N	0.201	2.493	4.001	39.913
TG/TBC_2_N	0.961	0.520	3.923	6.452
TG/TBN	0.697	0.717	3.521	11.039

Global hardness (*η*_G_) describes the resistance of a system toward electronic deformation or charge transfer; higher values generally indicate greater stability and lower chemical reactivity. In contrast, global softness (*β*_G_), which is inversely related to hardness, reflects the ability of a system to undergo electronic polarization and is often associated with enhanced reactivity.^52^ The chemical potential (*µ*) represents the escaping tendency of electrons from a system, where higher values indicate stronger electron-donating behavior. The electrophilicity index (*ω*) measures the ability of a system to accept electronic charge and is an important descriptor for understanding interfacial electronic behavior.^53,54^ As presented in Table 4, the investigated MBL systems show noticeable variations in their global reactivity descriptors, indicating distinct electronic characteristics. Among them, TBC_2_N exhibits the lowest hardness and relatively high chemical potential, reflecting its enhanced reactivity and greater tendency for charge transfer. In contrast, TG/TBC_2_N shows the highest hardness, suggesting comparatively greater electronic stability and reduced reactivity.

The electrophilicity index further highlights these trends. TBC_2_N displays the highest *ω* value, followed by TG, TG/TBC_2_N, and TG/TBN. The comparatively large electrophilicity values arise from the combined influence of chemical potential and global hardness. In particular, systems with smaller HOMO–LUMO gaps tend to exhibit enhanced electrophilic character due to increased charge-accepting ability. This behavior is consistent with previously reported theoretical studies on γ-SiC nanoflakes and tetragonal graphene-based nanostructures, where balanced softness and electrophilicity were linked to improved interfacial interaction and adsorption performance.

Overall, the calculated global descriptors indicate that all MBL systems exhibit moderate hardness values, suggesting a balance between structural stability and chemical reactivity. Compared with highly inert graphene-like systems, these values reflect enhanced reactivity, while still maintaining sufficient stability. Such a balance suggests that these materials may serve as useful model systems for studying interfacial electronic behavior and charge-transfer-driven applications.^48,54,55^

### 3.5 Optical properties (UV-vis analysis)

**Fig. 7:**
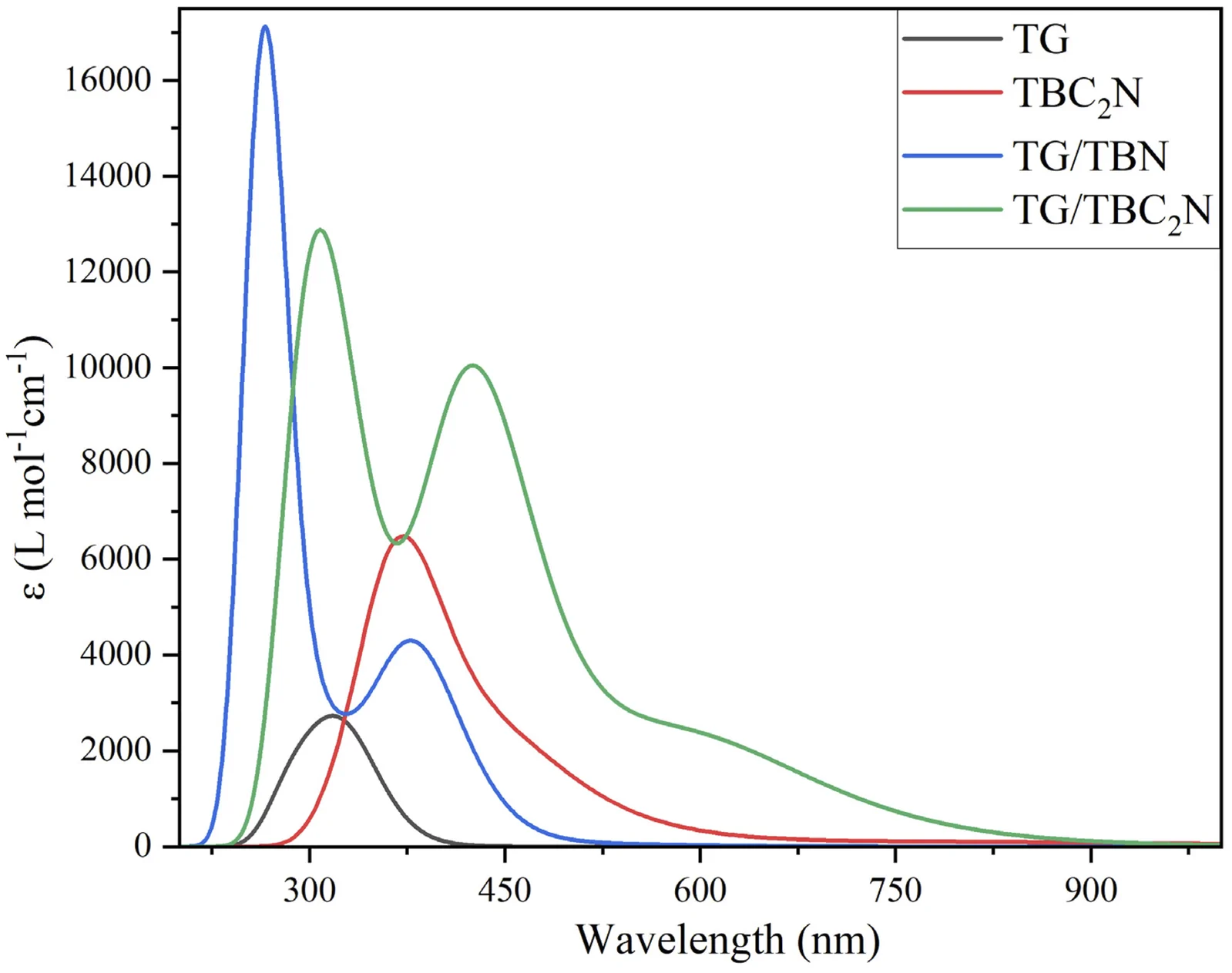
UV-vis spectra for all structures (molar absorption coefficient of the optimized geometries).

The optical properties of the investigated MBL systems were examined using time-dependent density functional theory (TD-DFT) to understand their electronic excitation behavior and optical response.^56^ The simulated UV-vis absorption spectra are presented in Fig. 7. In contrast, the corresponding maximum absorption wavelength (*λ*_max_), excitation energy (*E*_λ_), and oscillator strength (*f*) are summarized in Table 5. All investigated structures exhibit distinct absorption bands in the ultraviolet (UV) region, demonstrating their ability to absorb photons and undergo electronic excitation.^57^ The absorption profiles differ considerably among the four systems, indicating that the optical response is highly dependent on the atomic composition and bilayer configuration.

**Table 5:** Maximum absorption wavelength (*λ*_max_), excitation energy (*E*_λ_), and oscillator strength (*f*) of the investigated MBL systems

Structures	Wavelength (*λ*_max_) nm	Excitation energy (*E*_λ_) (eV)	Oscillator strength (*f*)
TG	329.9	3.759	0.0252
TBC_2_N	373.3	3.322	0.1369
TG/TBC_2_N	312.15	3.972	0.0268
TG/TBN	255.52	4.853	0.057

As shown in Fig. 7, pristine TG exhibits a characteristic absorption band centered at 329.9 nm. The TBC_2_N monolayer displays its maximum absorption at 373.3 nm, representing the longest absorption wavelength among the investigated systems. The TG/TBC_2_N bilayer exhibits a broad absorption band centered at 312.15 nm, whereas the TG/TBN bilayer shows a sharp absorption feature at 255.52 nm together with a relatively weak absorption tail extending toward longer wavelengths. These variations in the absorption profiles demonstrate that structural modification effectively alters the optical response of the proposed bilayer systems.

The excitation energy associated with the maximum absorption wavelength was determined using Planck's relation,^58^7
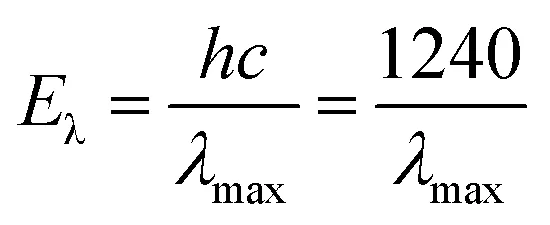
where *E*_λ_ is expressed in eV and *λ*_max_ is given in nm. Since excitation energy is inversely proportional to the absorption wavelength, longer absorption wavelengths correspond to lower excitation energies, whereas shorter wavelengths require higher excitation energies. Accordingly, the calculated excitation energies range from 3.322 eV for TBC_2_N to 4.853 eV for TG/TBN, while TG and TG/TBC_2_N exhibit excitation energies of 3.759 eV and 3.972 eV, respectively.

The oscillator strength represents the probability of an electronic transition induced by photon absorption.^59^ As summarized in Table 5, the TBC_2_N monolayer exhibits the highest oscillator strength (0.1369), indicating the greatest transition probability among the investigated systems. The TG/TBN bilayer shows the second-highest oscillator strength (0.0570), followed by TG/TBC_2_N (0.0268) and pristine TG (0.0252). The observed variation in oscillator strength demonstrates that the probability of electronic transitions is strongly dependent on the atomic arrangement of the investigated systems.

Overall, the calculated UV-vis spectra together with the TD-DFT-derived optical parameters reveal that the electronic excitation characteristics of the investigated MBL systems are strongly dependent on their structural configuration. Overall, the combined analysis of the absorption spectra, excitation energies, and oscillator strengths demonstrates that the optical response of the investigated MBL systems can be effectively tuned through structural modification, providing valuable insight into their excited-state properties.

### 3.6 Quantum theory of atoms in molecules (QTAIM)

**Fig. 8:**
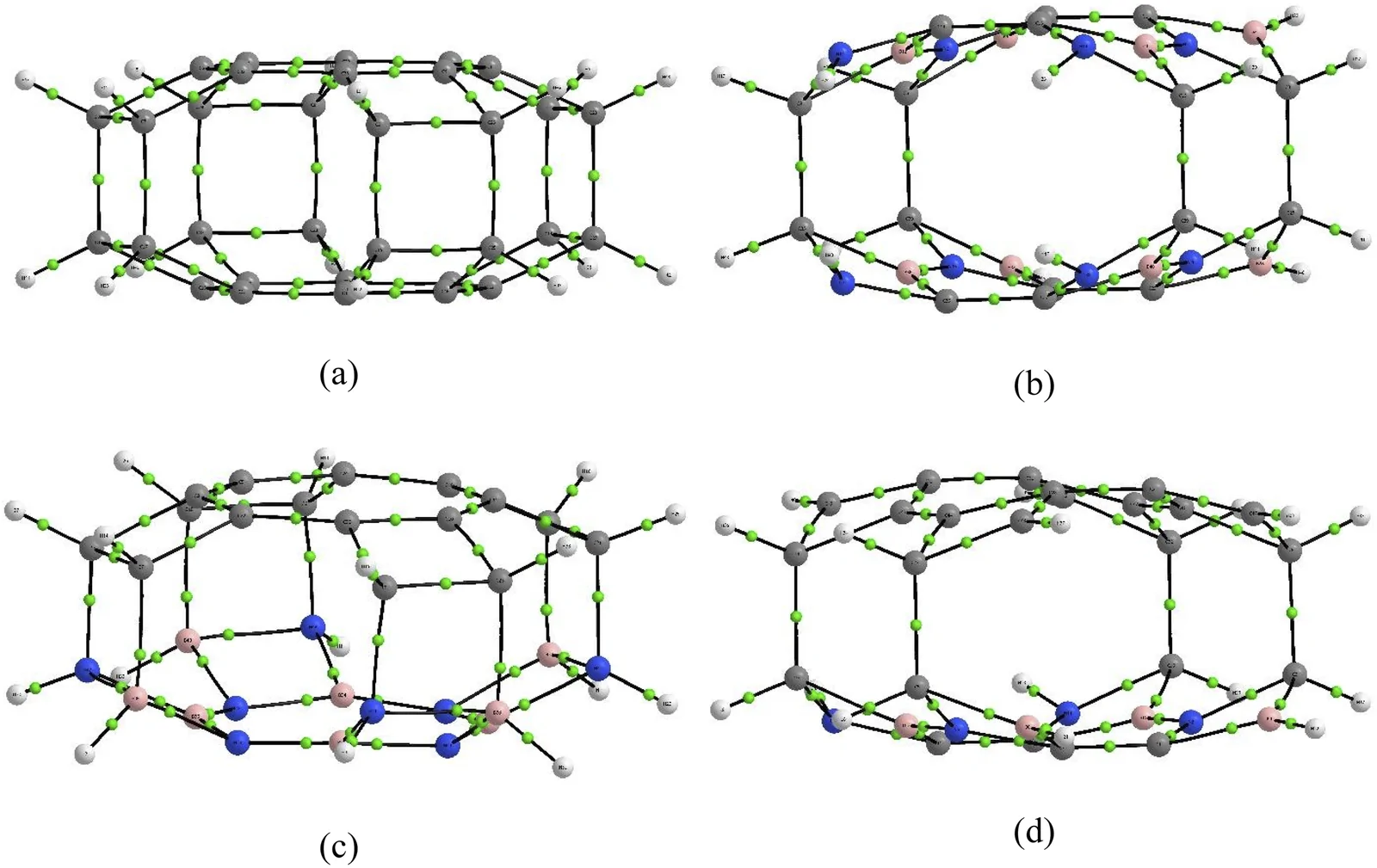
Molecular graph of the studied MBL structures (a) T-G, (b) T-BC_2_N, (c) T-G + T-BN, (d) T-G + T-BC_2_N.

The QTAIM, introduced by Bader and co-workers, provides a comprehensive framework for evaluating the nature and strength of interactions in molecular systems.^60,61^ In this work, QTAIM analysis was performed on the complete complex structures to gain deeper insight into the interactions between the two layers of the molecular structures.^62^ The presence of interlayer interactions was confirmed through the identification of bond critical points (BCPs),^63^ highlighted as green dots in Fig. 8. At these BCPs, descriptors such as electron density (*ρ*_e_^BC^), Laplacian of electron density (∇^2^*ρ*_e_^BC^), kinetic energy density (*G*_e_^BC^), potential energy density (*V*_e_^BC^), and total energy density (*H*_e_^BC^) were evaluated in Table 5 (ref. 64). The detection of BCPs indicates electron density sharing, confirming bond formation between the two layers of the molecular structures.

The interaction strength is reflected by *ρ*_e_^BC^ values, while ∇^2^*ρ*_e_^BC^ and *H*_e_^BC^ determine the bonding nature. For noncovalent interactions, *ρ*_e_^BC^ > 0.1 au corresponds to strong interactions such as hydrogen bonding or electrostatic forces, whereas *ρ*_e_^BC^< 0.1 au indicates weak van der Waals forces.^65,66^ As shown in Table 5, all studied complexes exhibit *ρ*_e_^BC^ > 0.1 au, confirming strong interactions between the two layers of the molecular structures. The sign of ∇^2^*ρ*_e_^BC^ and *H*_e_^BC^ further distinguishes the bonding type: both negative values indicate strong covalent interactions, both positive values correspond to weak noncovalent interactions, and ∇^2^*ρ*_e_^BC^ > 0 with *H*_e_^BC^ < 0 reflects partial covalency.^67,68^ In our case, the negative values of both parameters confirm strong covalent bonding between the two layers of the molecular structures Table 6.

**Table 6:** Electron density(*ρ*_e_), ∇^2^*ρ*_e_, electron's kinetic ED (*G*_e_), potential ED (*V*_e_), total ED(*H*_e_), −*G*_e_/*V*_e_, and ratios in au unit at BC points of intermolecular bonds in the structures

Structures	Bonds	*ρ*_e_^BC^	∇^2^*ρ*_e_^BC^	*G*_e_^BC^	*V*_e_^BC^	*H*_e_^BC^	−*G*_e_^BC^/*V*_e_^BC^
TG	C_27_–C_28_	0.190	−0.268	0.051	−0.170	−0.118	0.303
	C_14_–C_2_	0.190	−0.268	0.051	−0.170	−0.118	0.303
	C_25_–C_26_	0.190	−0.267	0.051	−0.170	−0.118	0.303
	C_15_–C_3_	0.190	−0.268	0.051	−0.170	−0.118	0.303
	C_20_–C_8_	0.190	−0.268	0.051	−0.170	−0.118	0.303
	C_33_–C_34_	0.190	−0.267	0.051	−0.170	−0.118	0.303
	C_17_–C_5_	0.190	−0.268	0.051	−0.170	−0.118	0.303
	C_37_–C_38_	0.190	−0.268	0.051	−0.170	−0.118	0.303
T-BC_2_N	C_5_–C_29_	0.191	−0.281	0.049	−0.169	−0.120	0.2921
	C_3_–C_27_	0.191	−0.281	0.049	−0.169	−0.120	0.292
	C_15_–C_39_	0.196	−0.298	0.051	−0.177	−0.126	0.290
	C_9_–C_33_	0.196	−0.298	0.051	−0.177	−0.126	0.290
TG/T-BN	C_7_–B_39_	0.140	−0.160	0.079	−0.198	−0.119	0.399
	C_14_–B_40_	0.140	−0.159	0.079	−0.199	−0.119	0.400
	C_23_–B_38_	0.140	−0.159	0.079	−0.199	−0.119	0.400
	C_12_–B_37_	0.140	−0.159	0.079	−0.199	−0.119	0.400
	C_6_–N_48_	0.187	−0.213	0.080	−0.213	−0.133	0.400
	C_9_–N_44_	0.187	−0.213	0.080	−0.213	−0.132	0.375
	C_17_–N_47_	0.188	−0.214	0.080	−0.213	−0.133	0.375
	C_21_–N_41_	0.188	−0.214	0.080	−0.213	−0.133	0.375
TG/T-BC_2_N	C_3_–C_26_	0.200	−0.319	0.052	−0.183	−0.131	0.282
	C_5_–C_27_	0.200	−0.319	0.052	−0.183	−0.131	0.282
	C_9_–C_29_	0.202	−0.327	0.052	−0.186	−0.134	0.280
	C_15_–C_32_	0.202	−0.327	0.052	−0.186	−0.134	0.280

The bond character was further validated using the −*G*_e_^BC^/*V*_e_^BC^ ratio. Ratios <0.5 indicate covalent interactions, values >1 correspond to noncovalent interactions, and intermediate values (0.5–1) suggest partial covalency.^46^ For all studied MBL structures, the ratios are <0.5, reinforcing the covalent nature of the interactions between the two layers (L1 and L2) of the molecular structures. QTAIM and RDG analyses probe different aspects of interlayer interactions. To gain deeper insight into the nature of the interlayer interactions, the QTAIM descriptors were analyzed collectively, including the electron density at the bond critical point (*ρ*_e_^BC^), its Laplacian (∇^2^*ρ*_e_^BC^), the total energy density (*H*_e_^BC^), and the ratio −*G*_e_^BC^/*V*_e_^BC^. The combined behavior of these parameters consistently suggests that the interlayer interactions possess a predominant covalent character. QTAIM identifies localized bond critical points associated with direct covalent interlayer bonding, whereas RDG analysis visualizes weaker diffuse interactions distributed across the interfacial regions. Consequently, the covalent interlayer linkages revealed by QTAIM coexist with weaker π–π stacking and dispersive interactions identified through RDG analysis. Overall, the QTAIM parameters consistently demonstrate covalent bonding in all MBL structures, with variations in bond strength depending on the heteroatom substitution.

### 3.7 Reduced density gradient (RDG) analysis

**Fig. 9:**
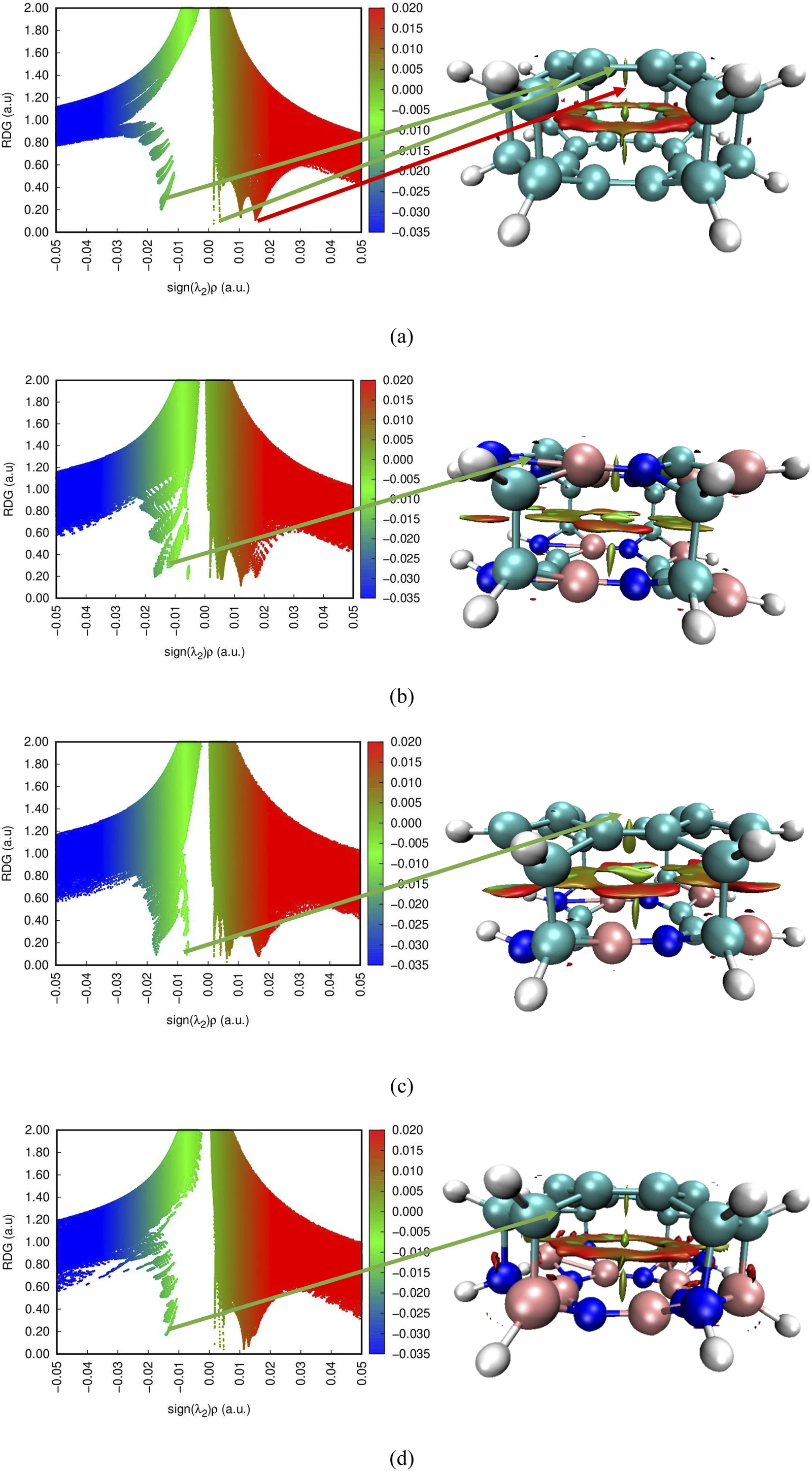
RDG scatter plot and isosurfaces of the complex structures (a) TG, (b) TBC_2_N, (c) TBC_2_N/TBN, (d) TG/TBN.

The RDG method enables a graphical representation of noncovalent interactions in molecular systems, providing more intuitive insights compared to QTAIM analysis.^39,61^ The non-covalent interaction (NCI) index utilizes two scalar fields, the electron density (*ρ*(*r*)) and the RDG, to depict local bonding features, defined as:^69,70^8
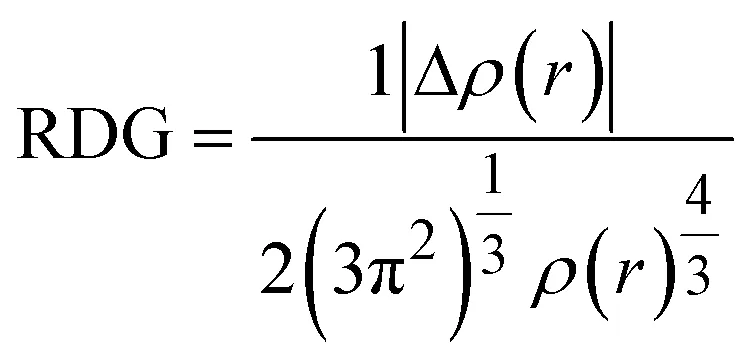
Here, the electron density *ρ*(*r*) and its derivative Δ*ρ*(*r*) are employed to simplify the NCI index, while (*λ*_2_)*ρ* represents the second eigenvalue of the electron density Hessian matrix, providing crucial insight into both the type and strength of interactions. Fig. 9 presents the gradient isosurfaces of the complexes along with scatter plots in the red–green–blue spectrum, illustrating interaction strength. In these plots, blue regions correspond to strong attractive forces, such as hydrogen bonding or dipole–dipole interactions, red indicates steric repulsion or overlapping noncovalent regions, and green represents weak attractive forces, including van der Waals interactions and π–π stacking.^70,71^ In all studied structures, no prominent blue regions are observed between the two layers, suggesting the absence of strong attractive noncovalent interactions. Green isosurfaces indicate weak dispersive interactions, while red regions correspond to steric repulsion between the layers.

The interaction character can also be inferred from the sign(*λ*_2_)*ρ* values: positive values (>0) indicate repulsion, negative values (<0) indicate attraction, and values near zero (≈0) correspond to weak interactions.^72^Fig. 9 shows that all MBL structures display a distinct green spike between −0.02 and −0.01 a.u., indicating significant attractive interactions between the two layers, consistent with the QTAIM analysis.

## 4 Conclusion

In this computational study, we systematically investigated the viability and properties of four bilayer systems, pristine MBL-TG, MBL-TBC_2_N, and the hetero-bilayers MBL-TG/TBN and MBL-TG/TBC_2_N, using dispersion-corrected density functional theory calculations. Our findings confirm that all four MBLs are dynamically and energetically stable, as evidenced by the complete absence of imaginary vibrational frequencies and significant negative cohesive energies. The electronic properties are found to be highly tunable *via* heteroatom substitution and stacking. All MBLs are semiconductors, with energy gaps ranging from 0.401 eV to 1.921 eV. This tunability extends to their chemical reactivity; MBL-TBC_2_N was identified as the most reactive, possessing the lowest global hardness and highest electrophilicity index. Furthermore, analysis of ESP maps and FMOs identified distinct electron-rich (N sites) and electron-poor (B sites) regions, highlighting preferential sites for molecular interactions. Optically, the MBLs exhibit strong absorption in the UV-vis spectrum. Heterostructure formation induces a notable redshift and broadened absorption into the visible range, demonstrating strong interlayer electronic coupling. QTAIM analysis unambiguously demonstrates strong covalent character in interlayer bonding, while complementary RDG visualizations reveal additional π–π* stacking and van der Waals contributions, resulting in stable vertical stacking without structural collapse. These findings illustrate that strategic heteroatom substitution and vertical heterostructure of tetragonal carbon frameworks offer a powerful route to tailor electronic, optical, and chemical properties at the molecular scale. The combination of tunable semiconducting gaps, interlayer electronic coupling, and surface polarization demonstrates that these tetragonal molecular bilayers may serve as useful model platforms for future investigations of low-dimensional optoelectronic and interfacial electronic materials. Further adsorption, transport, and device-level studies will be necessary to evaluate their suitability for practical sensing or catalytic applications.

## Author contributions

Abu Talha: data curation, formal analysis, investigation, visualization, writing – original draft, Mohsina Faria Mou: formal analysis, visualization, writing – original draft, Provati Rahman: data curation, visualization, writing – original draft, Abdullah Al Roman: resources, software, validation, writing – review and editing, Debashis Roy and Mohammad Tanvir Ahmed: conceptualization, methodology, project administration, supervision, writing – review and editing.

## Conflicts of interest

The authors of this manuscript have no conflicts of interest to declare.

## Supplementary Material

RA-016-D6RA04653C-s001

## Data Availability

Data associated with this manuscript will be shared upon reasonable request. Supplementary information (SI): additional computational data using the LANL2DZ basis set, including figures and tables supporting the structural, electronic, vibrational, and bonding analyses presented in the manuscript. See DOI: https://doi.org/10.1039/d6ra04653c.
