# Supplementary material for: Comparative study of the structural and optoelectronic properties of covalently bonded T-graphene-like bilayer nanoflakes: an ab initio investigation
Source: RSC Adv. 2026 Aug 11;16(40):46961–77. doi: 10.1039/d6ra04653c (PMC13460251; doi:10.1039/d6ra04653c)
Supplement: Supplementary file 1 [file RA-016-D6RA04653C-s001.pdf]

Supplementary Information (SI) for RSC Advances.
This journal is © The Royal Society of Chemistry 2026

Supplementary Figures Using LANL2DZ Basis Set

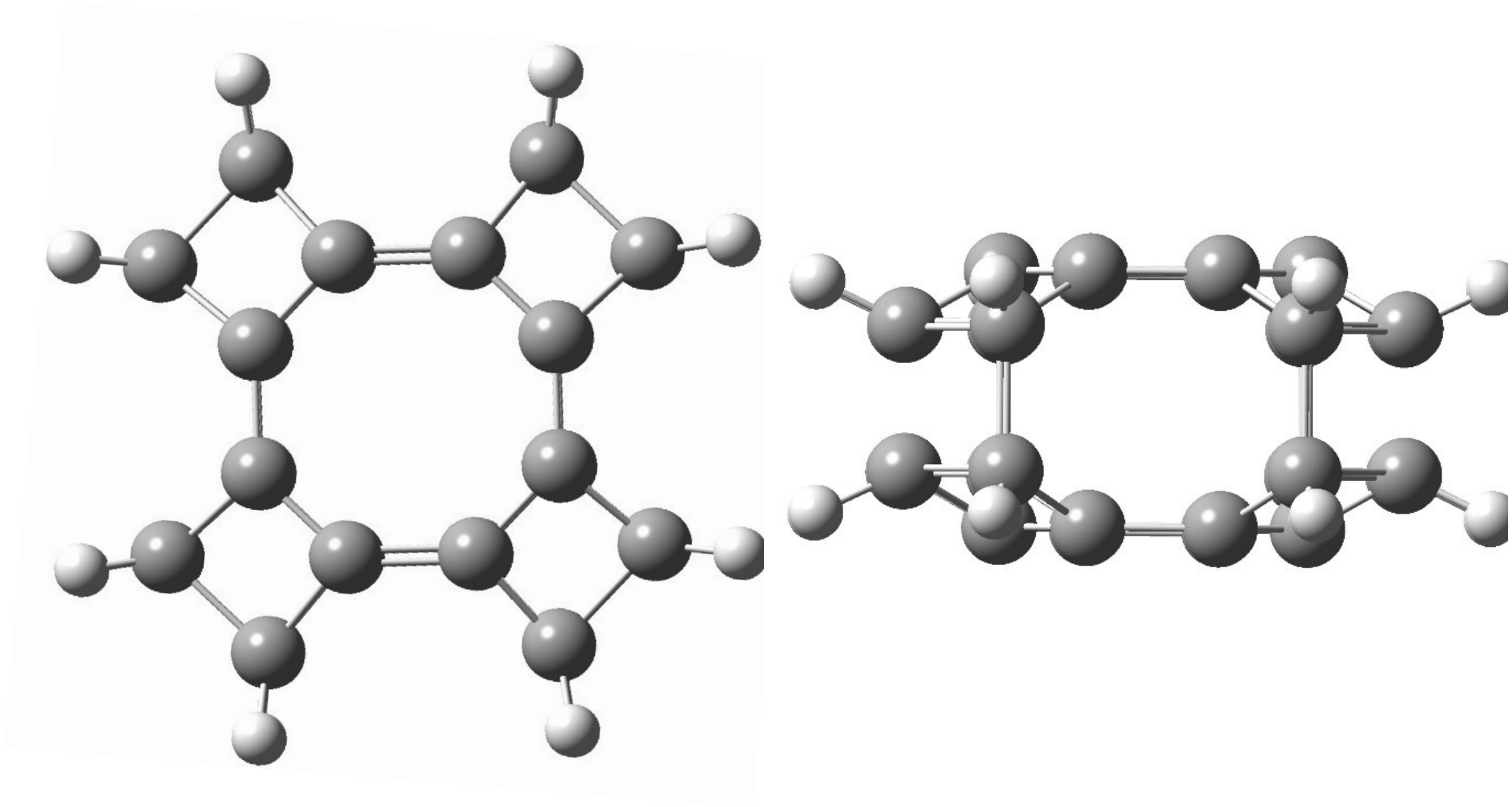

(a)

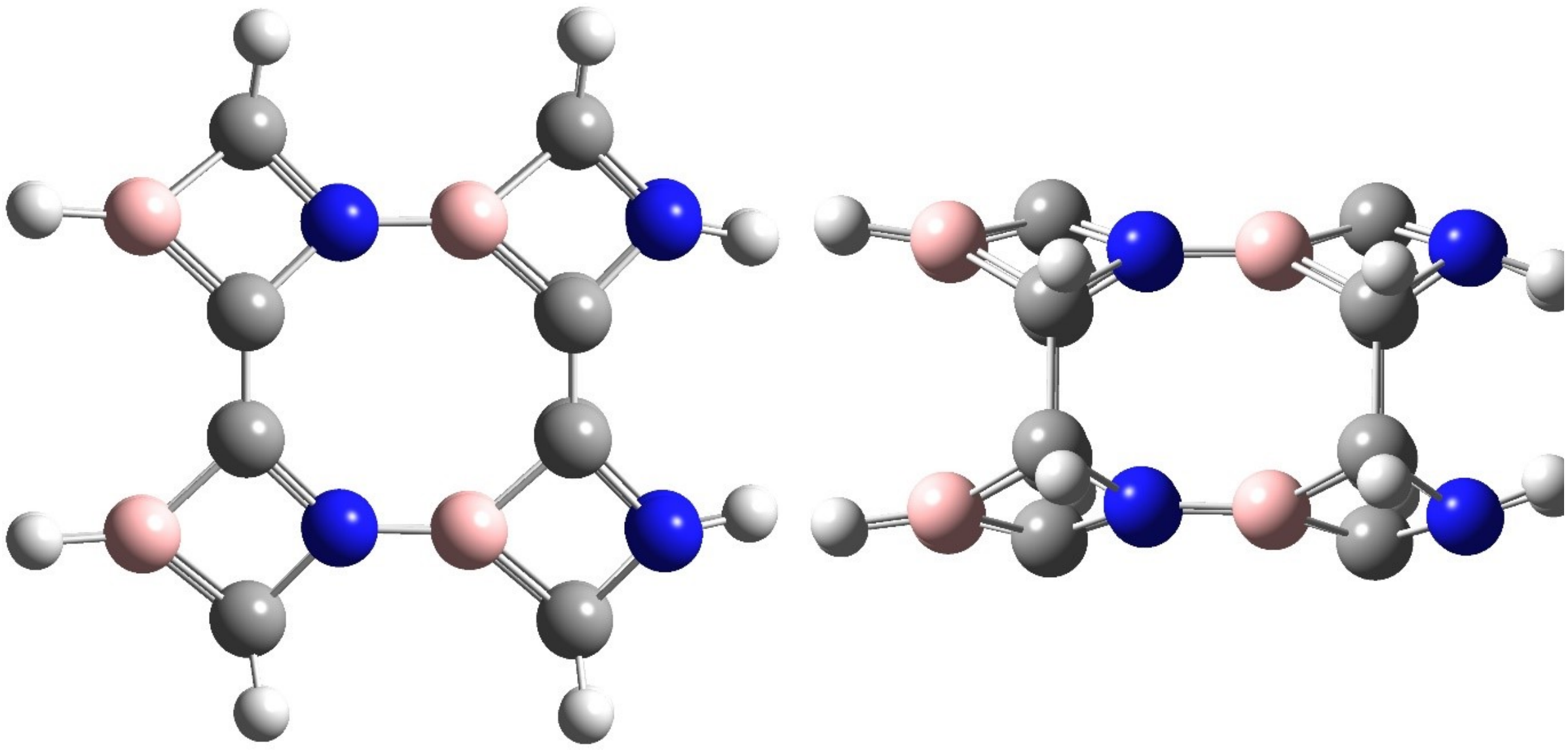

(b)

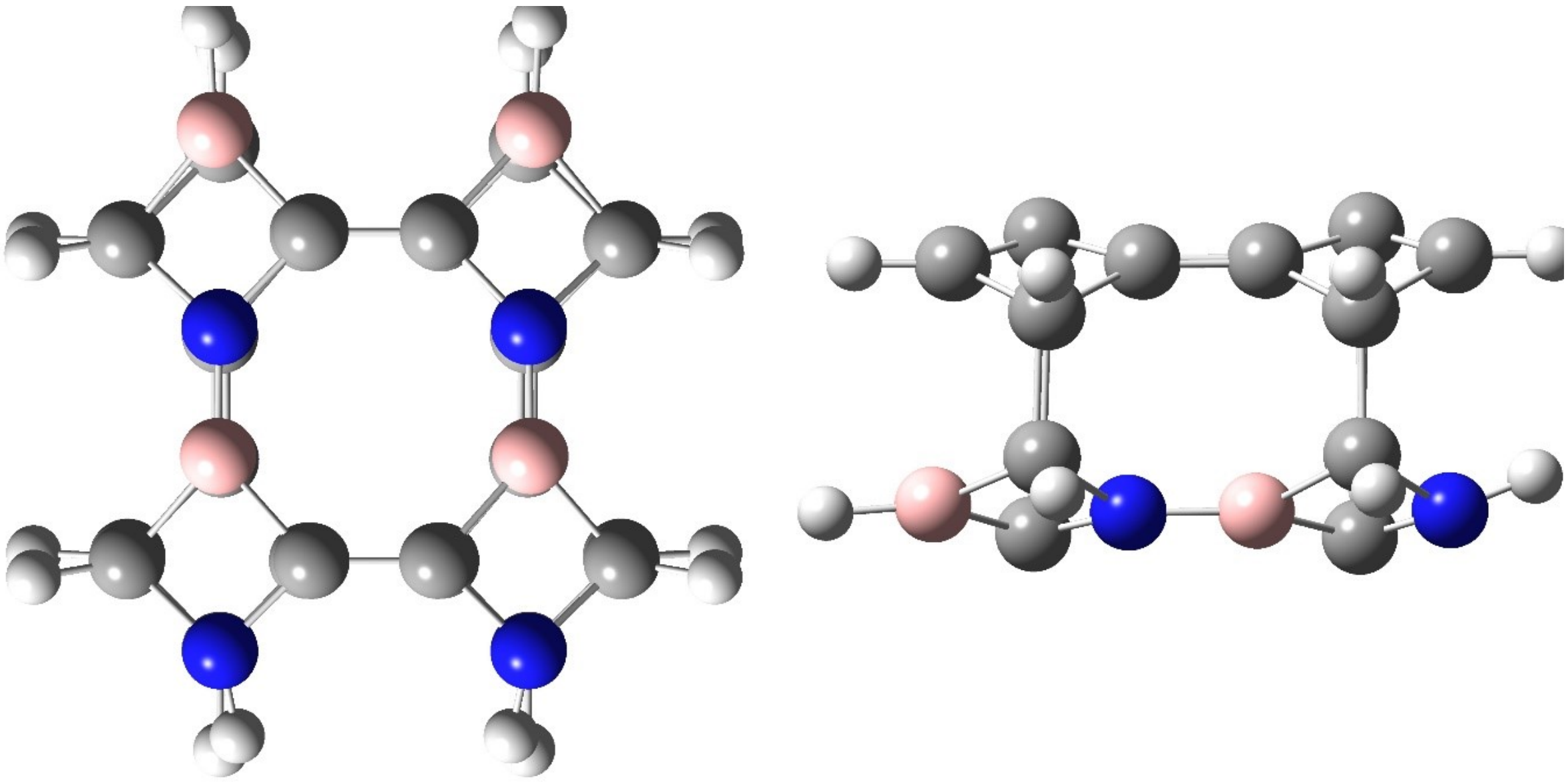

(c)

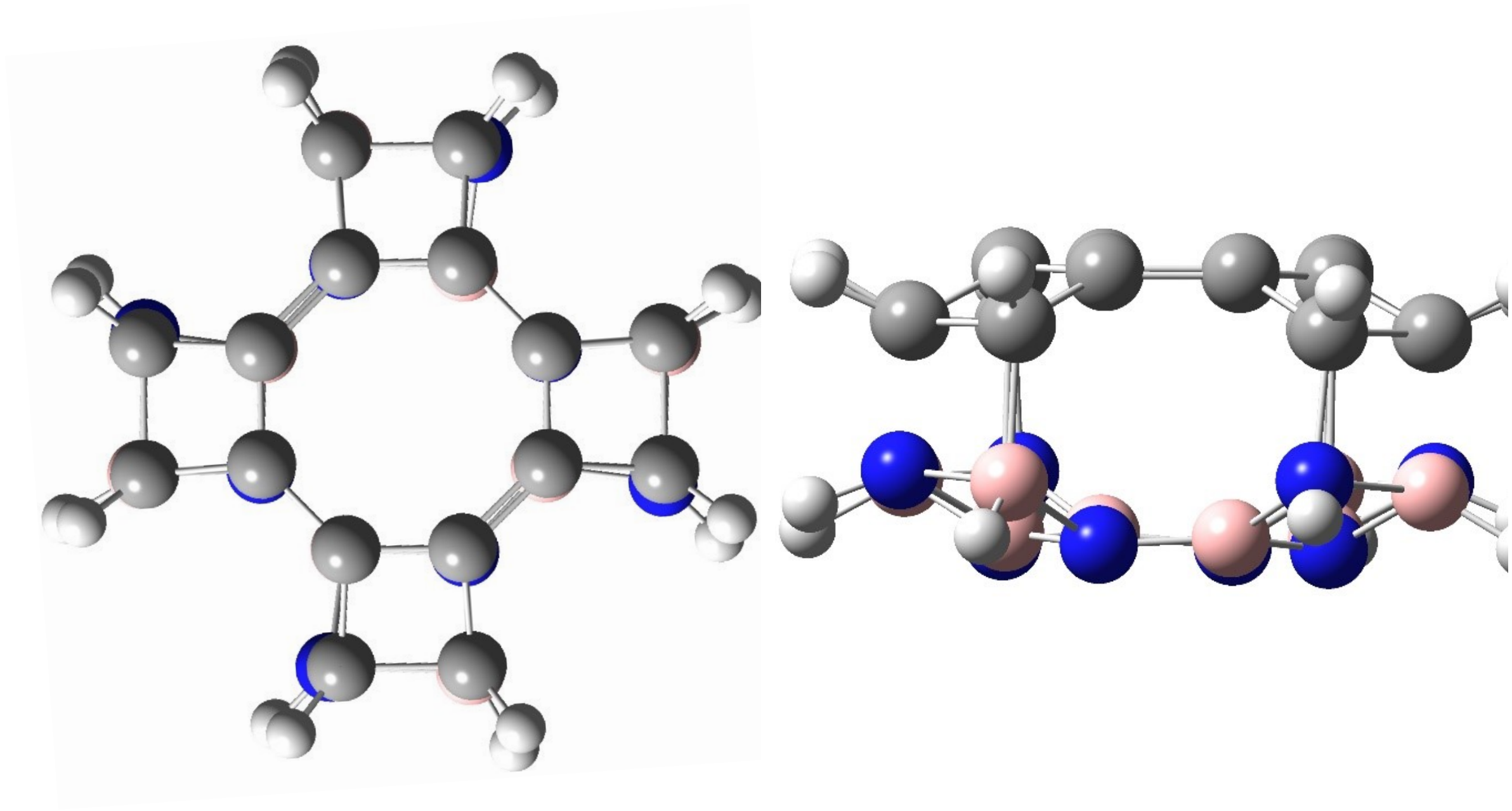

(d)

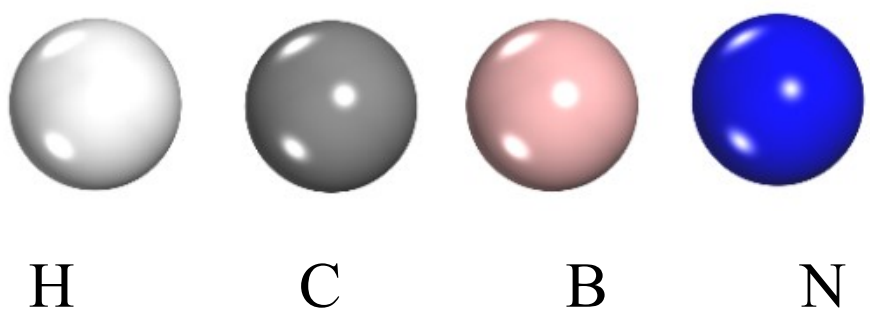

Figure 1: Optimized geometries of the MBL systems after full structural relaxation: (a) TG, (b) TBC_2N, (c) TG/TBC_2N, (d) TG/TBN.

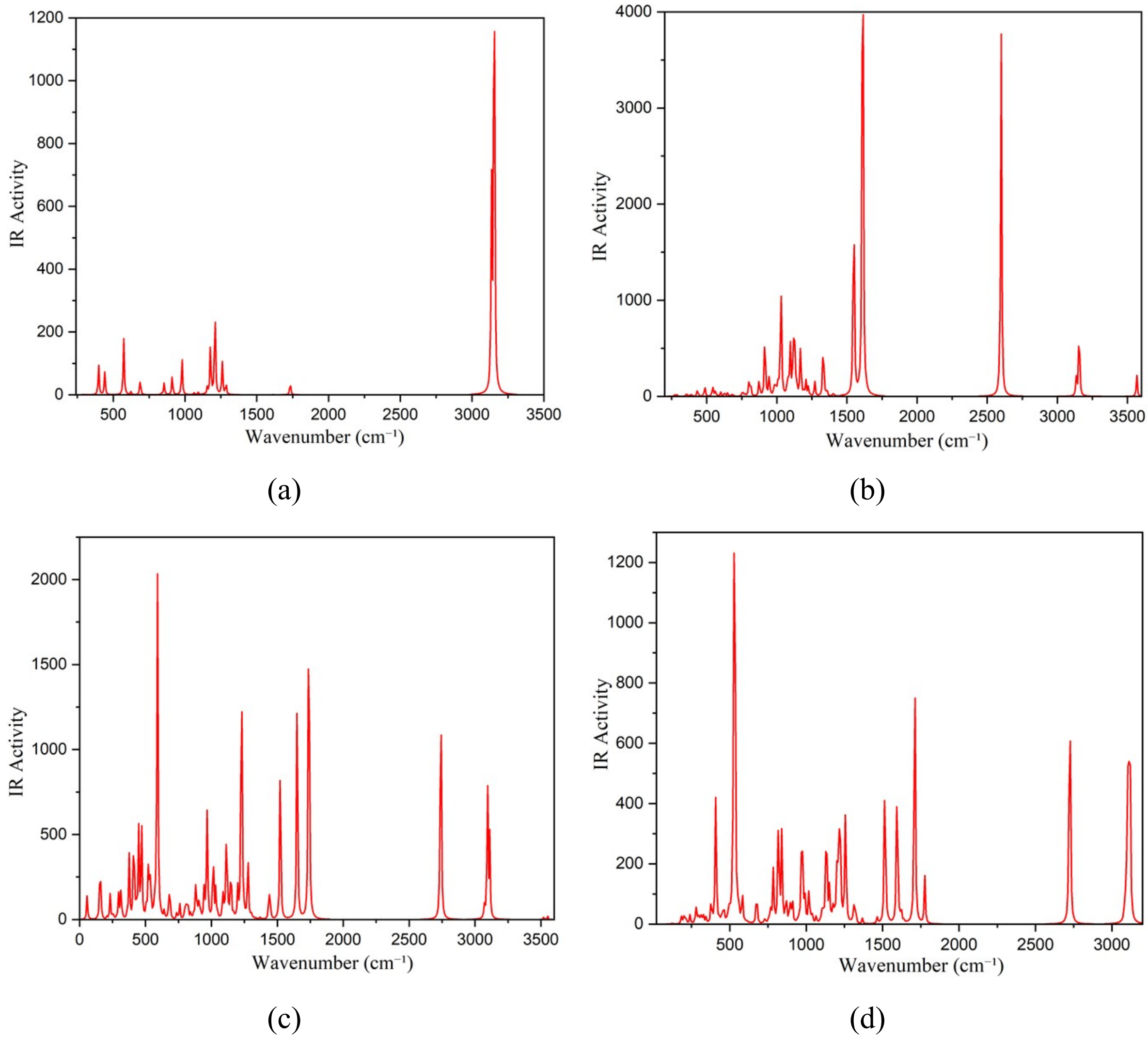

Figure 2: Infrared (IR) spectra of MBL systems: (a) TG, (b) TG/TBN, (c) TBC_2N, (d) TG/TBC_2N.

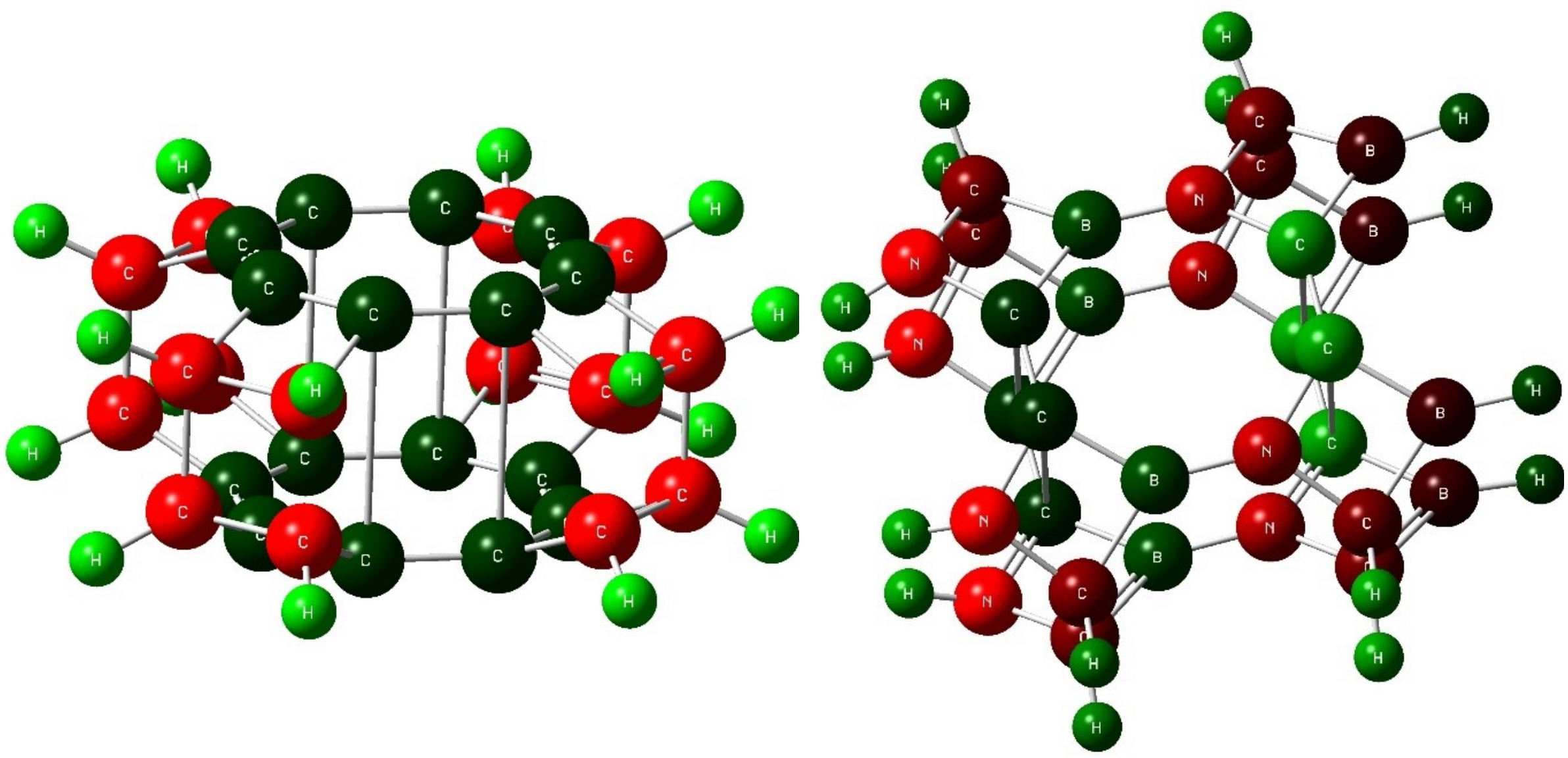

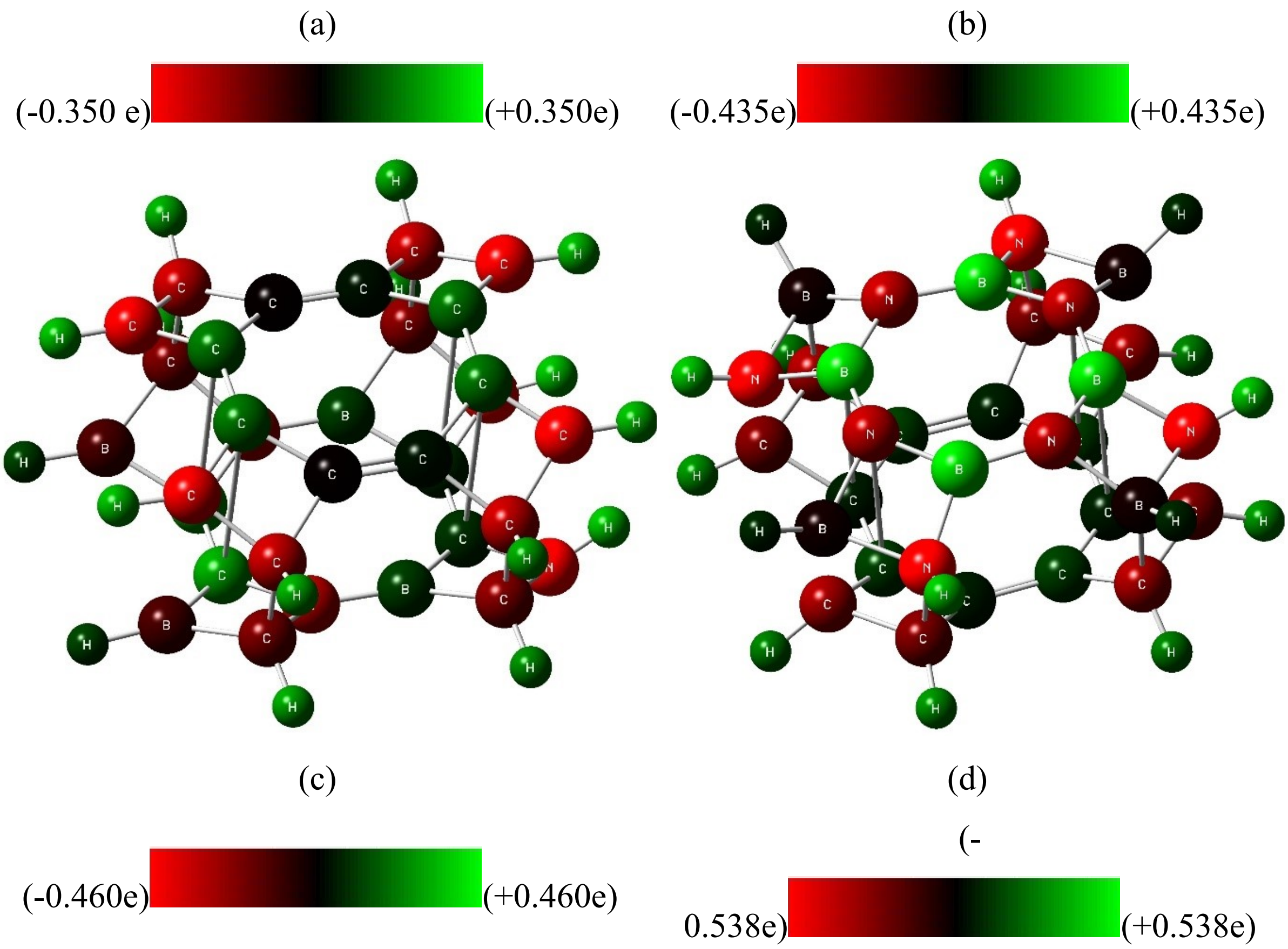

Figure 3: Distribution of Mulliken charges in the optimized MBL structures: (a) TG, (b) TBC_2N, (c) TG/TBC_2N, (d) TG/TBN.

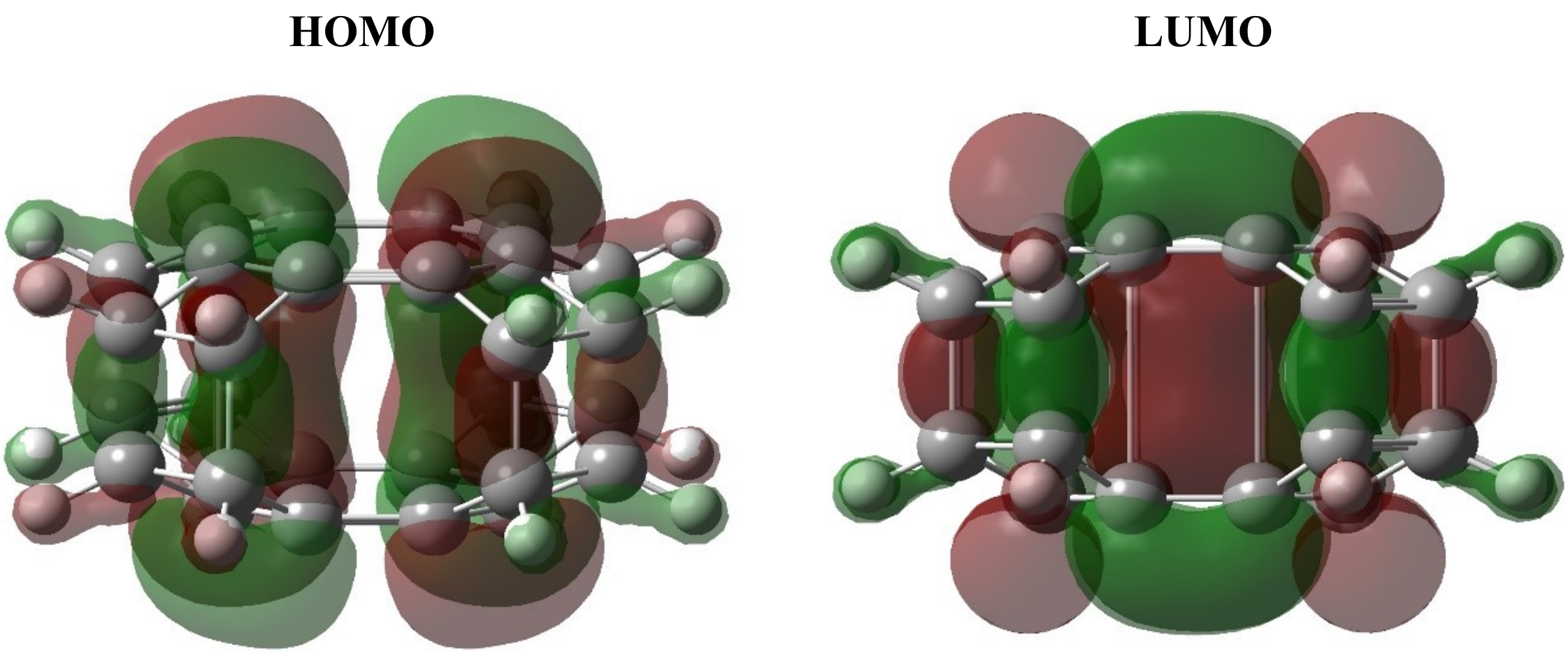

(a)

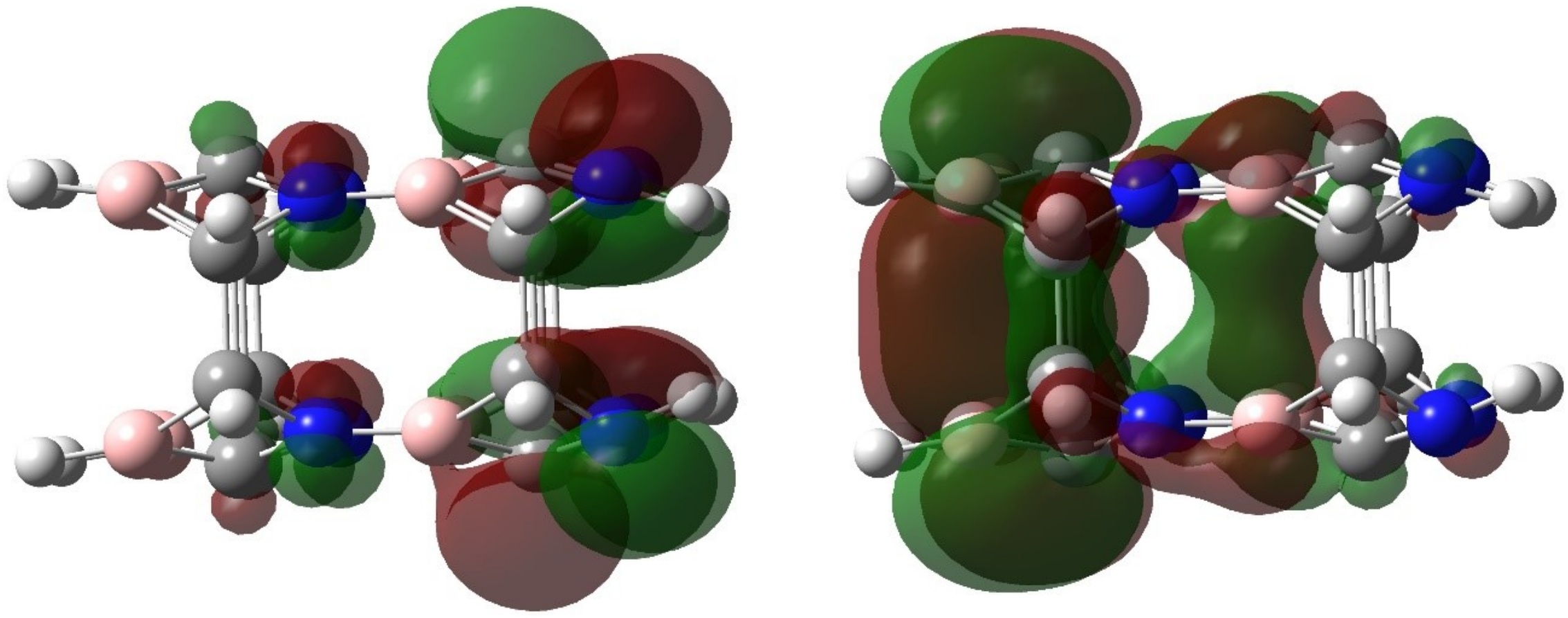

(b)

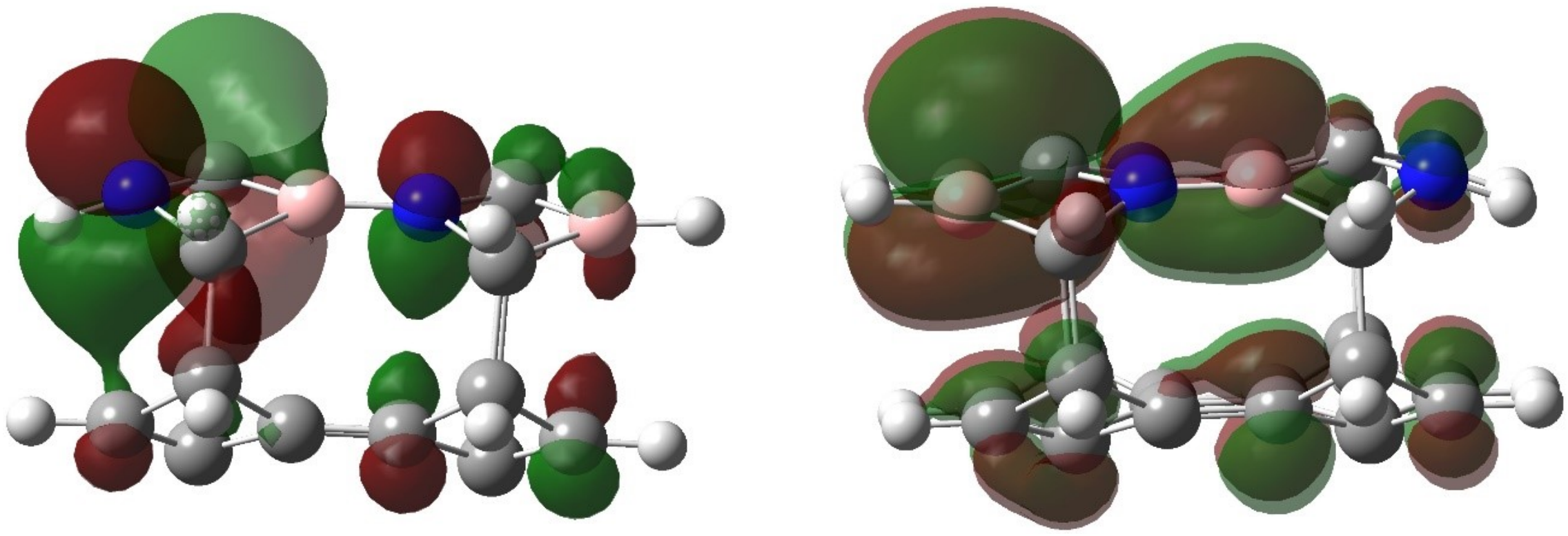

(c)

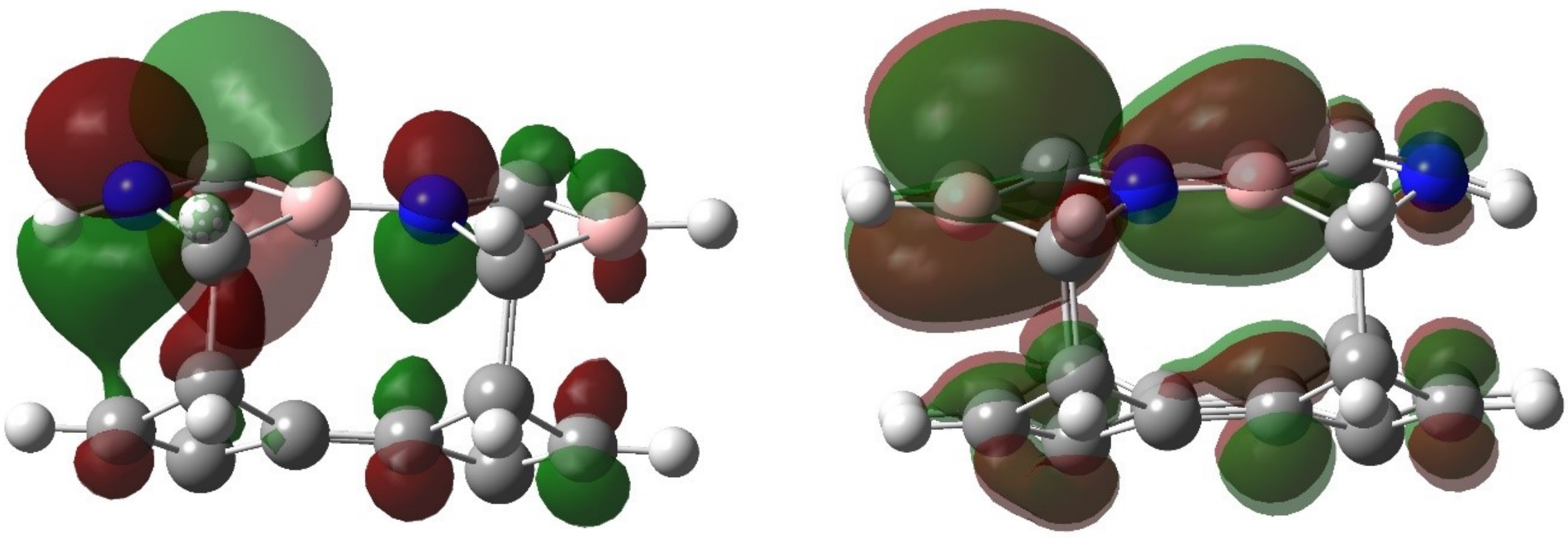

(d)

Figure 4: HOMOs and LUMOs of studied MBL structures: (a) TG, (b) TBC_2N, (c) TG/TBN, (d) TG/TBC_2N.

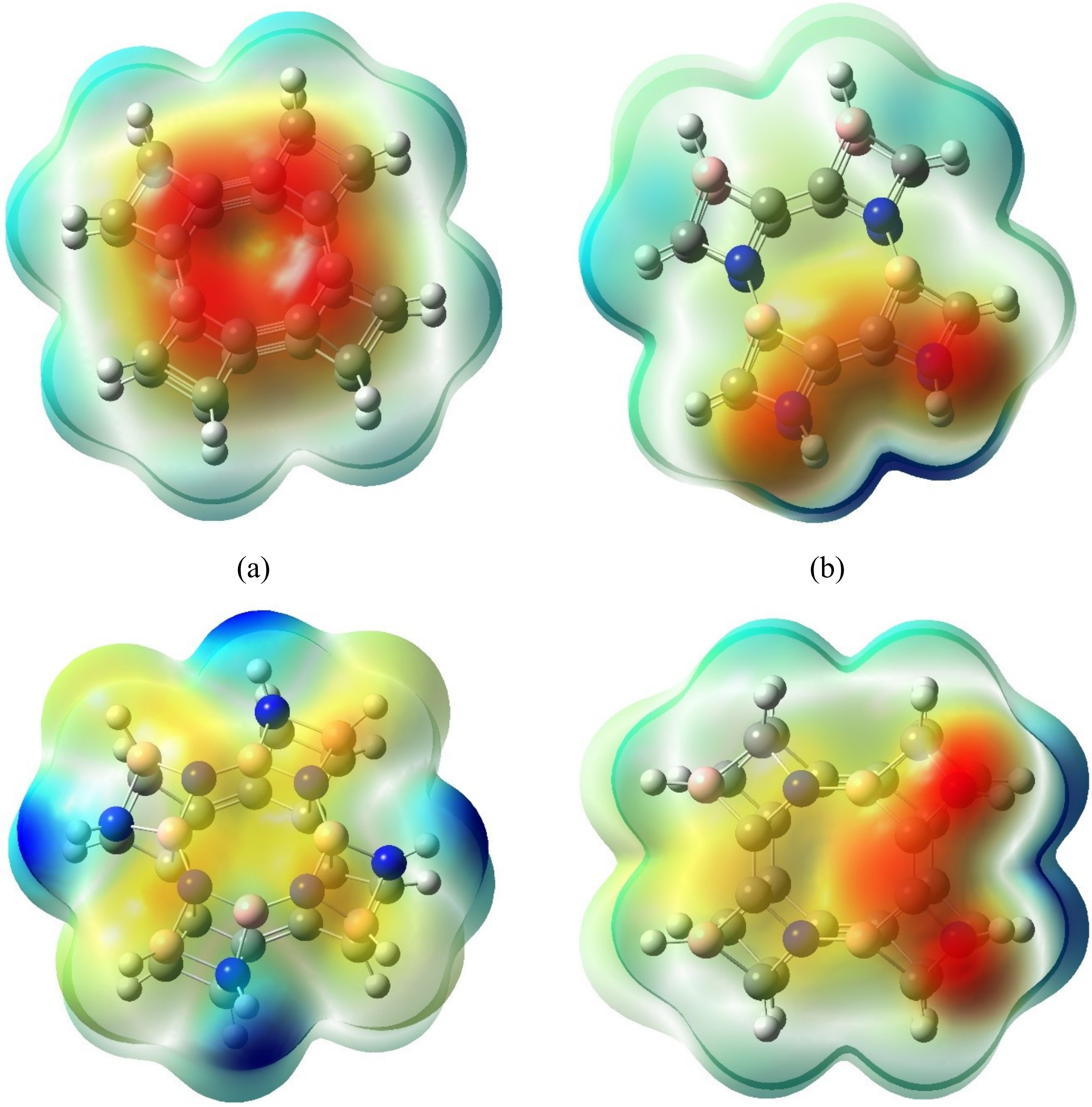

(a) (b) (c) (d)

Figure 5: Electrostatic potential map of studied MBL structures : (a) TG (-4.739e-2 to 4.739e-2), (b) TBC_2N (-5.125e-2 to 5.125e-2), (c) TG/TBN (-4.739e-2 to 4.739e-2), (d) TG/TBC_2N (-4.424e-2 to 4.424e-2).

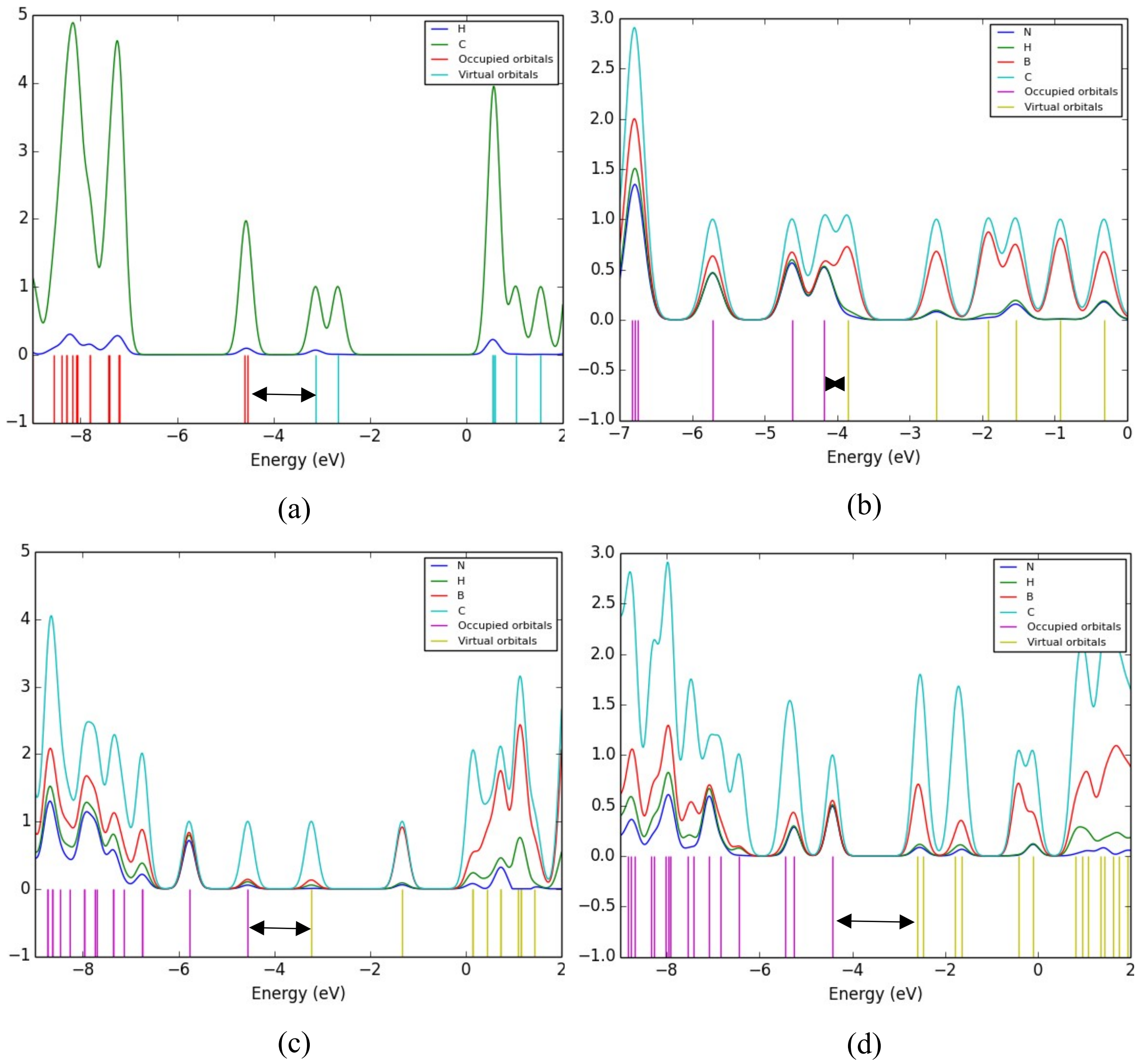

Figure 6: Partial density of state of studied MBL structures: (a) TG, (b) TBC_2N, (c) TG/TBN (d) TG/TBC_2N.

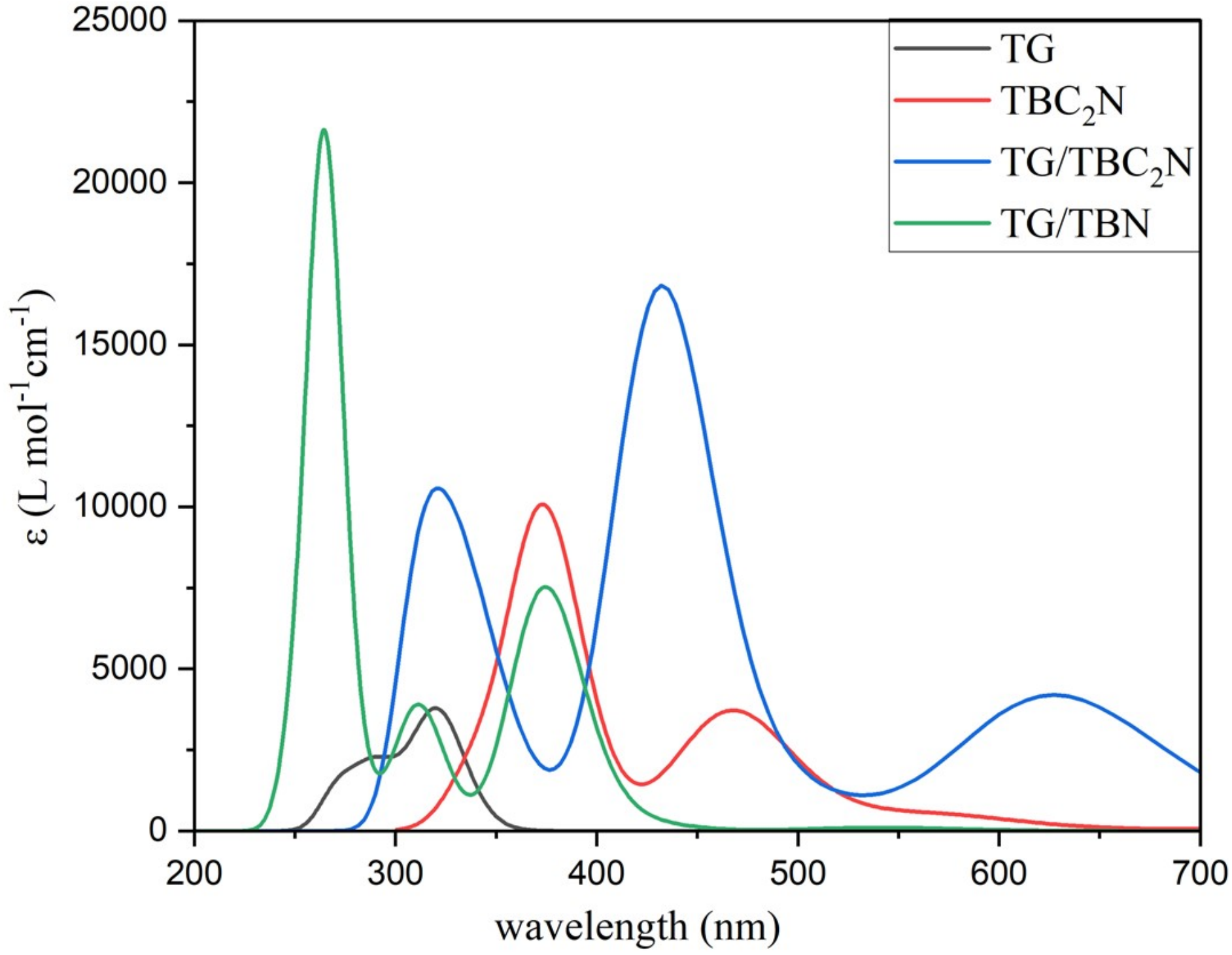

Figure 7: UV-Vis for all structures (Molar absorption coefficient of the optimized geometries.

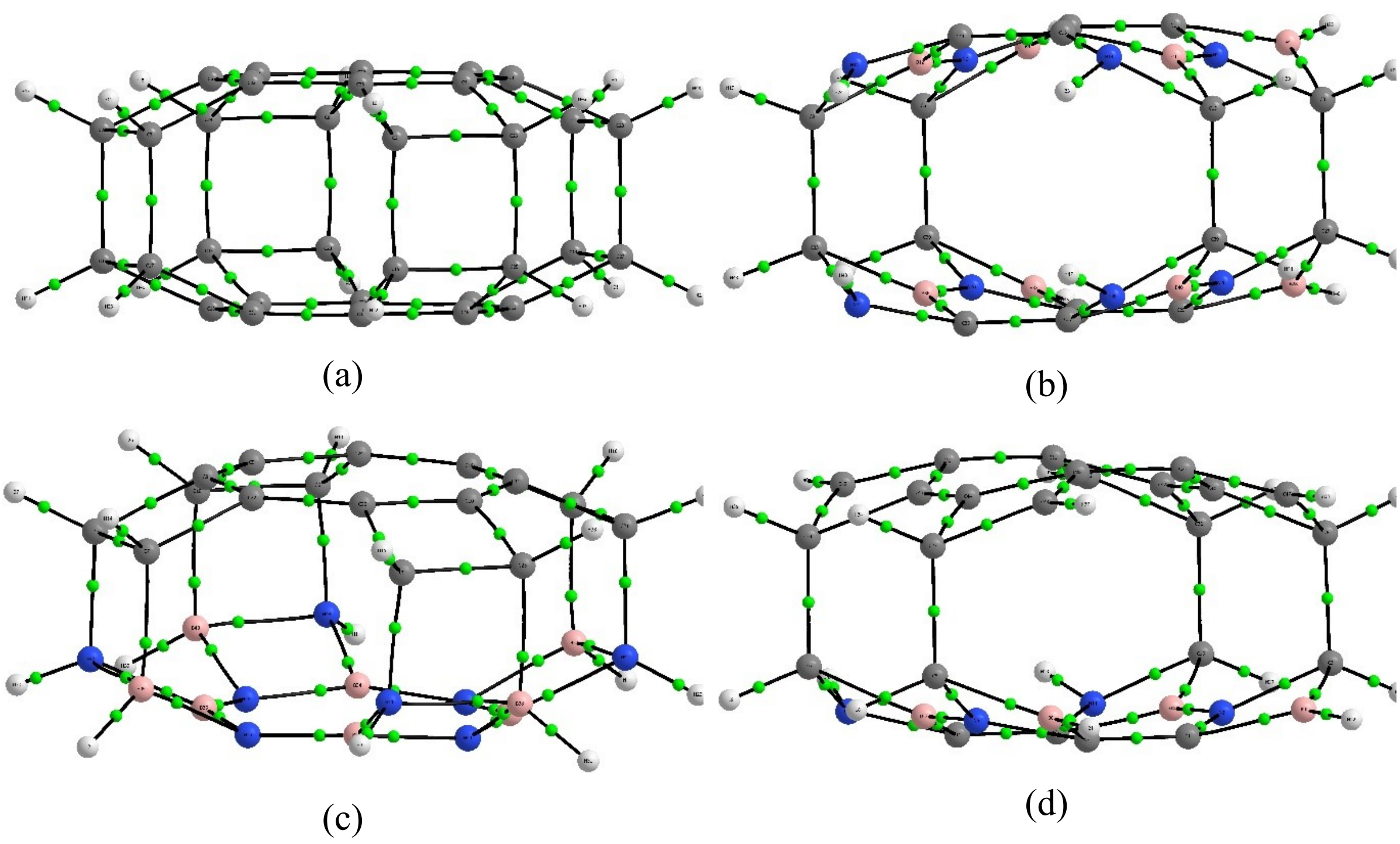

Figure 8: Molecular graph of the studied MBL structures (a) T-G,(b) T-BC_2N, (c) T-G+T-BN, (d) T-G+T-BC_2N.

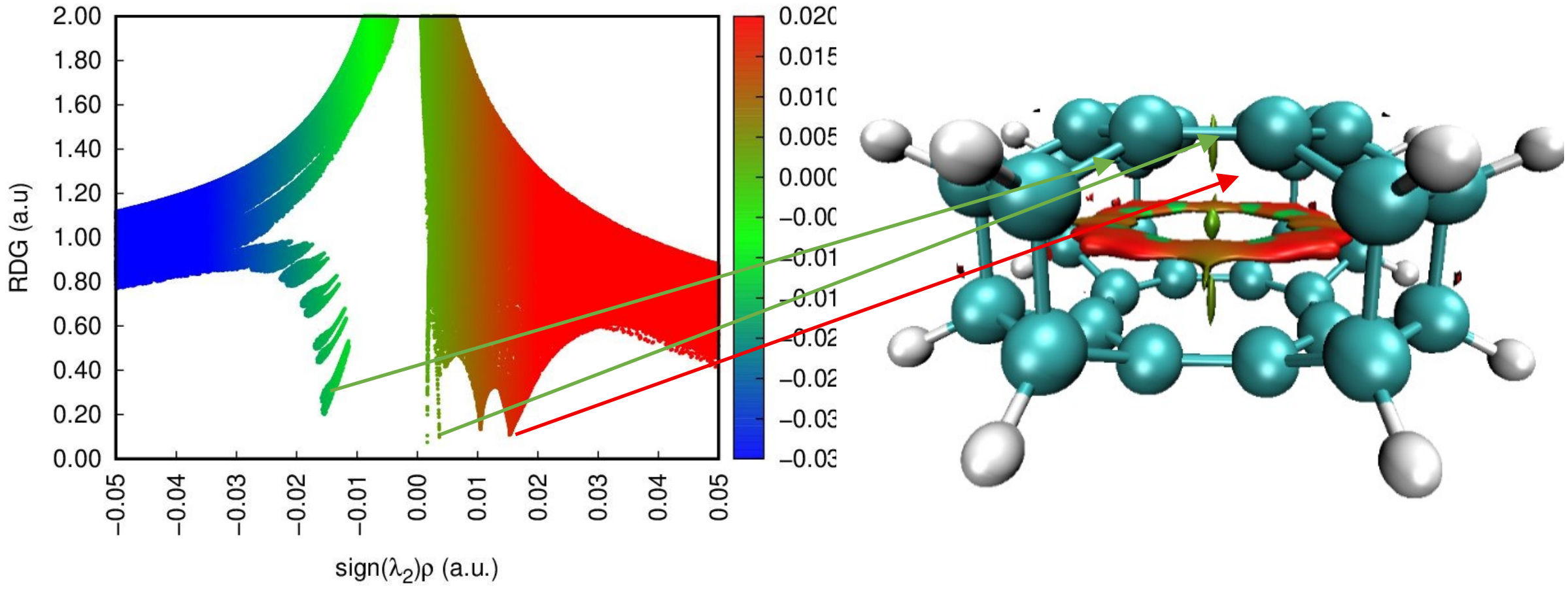

(a)

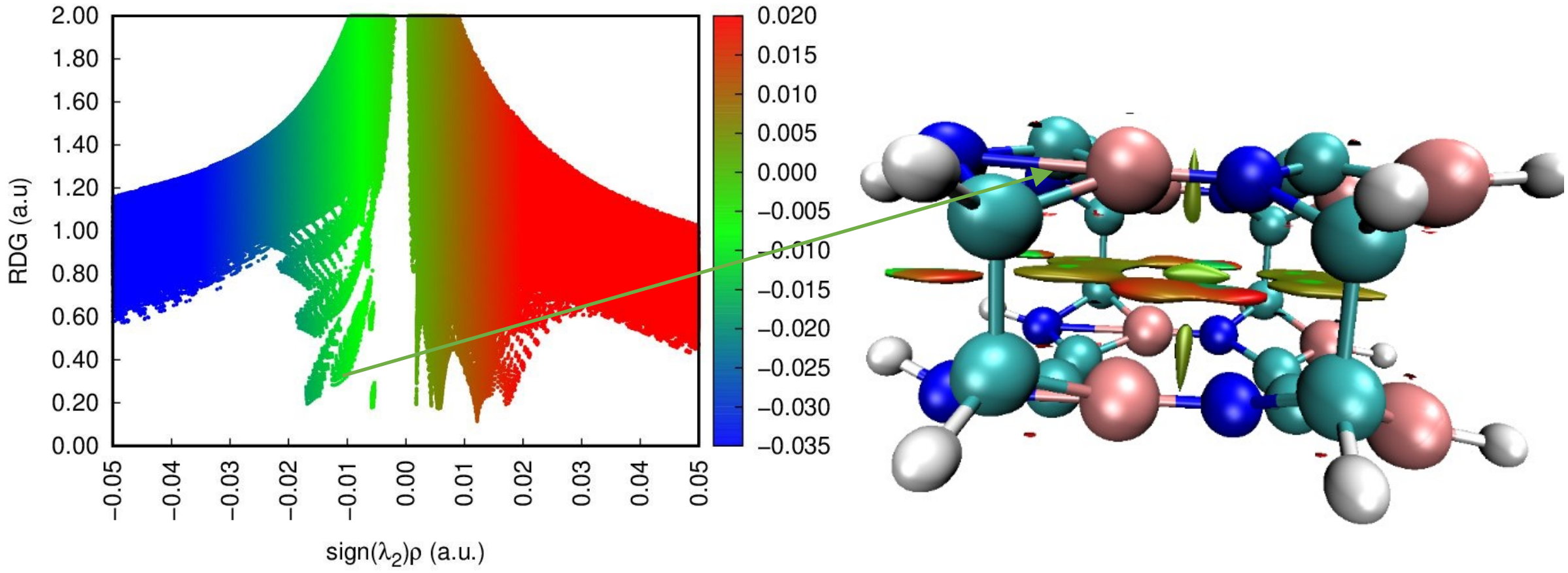

(b)

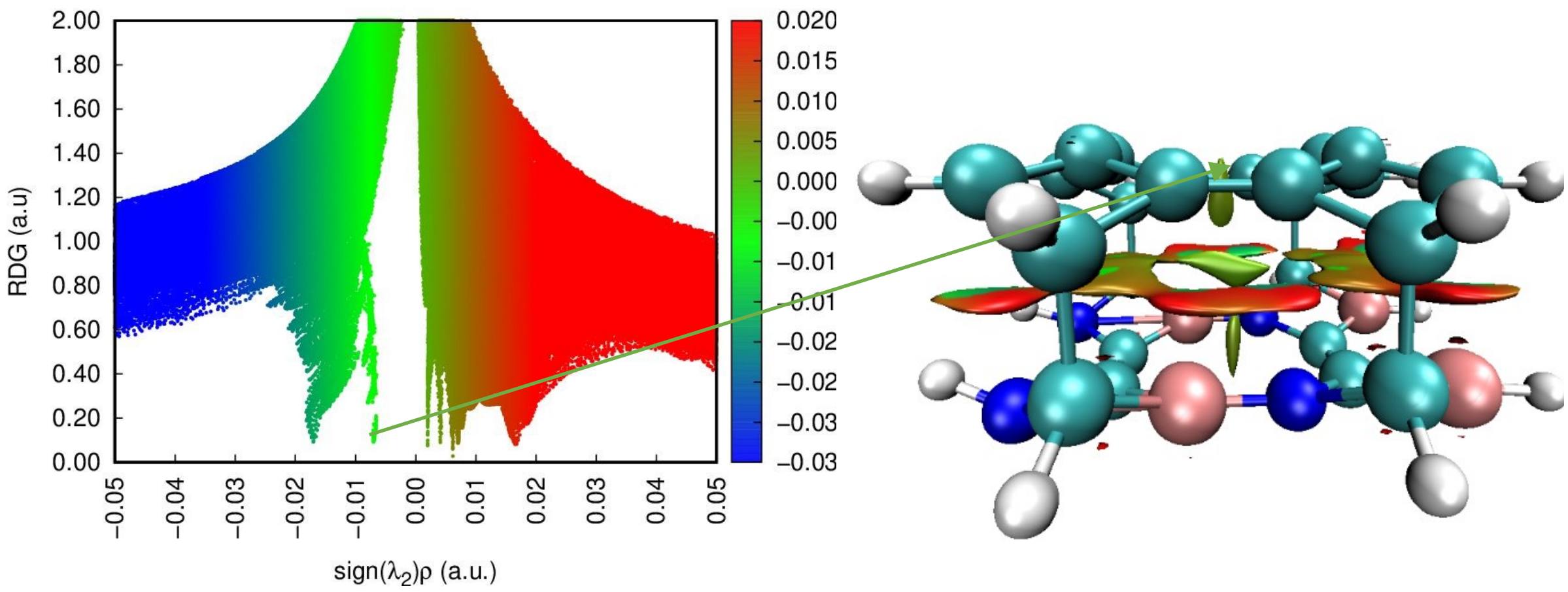

(c)

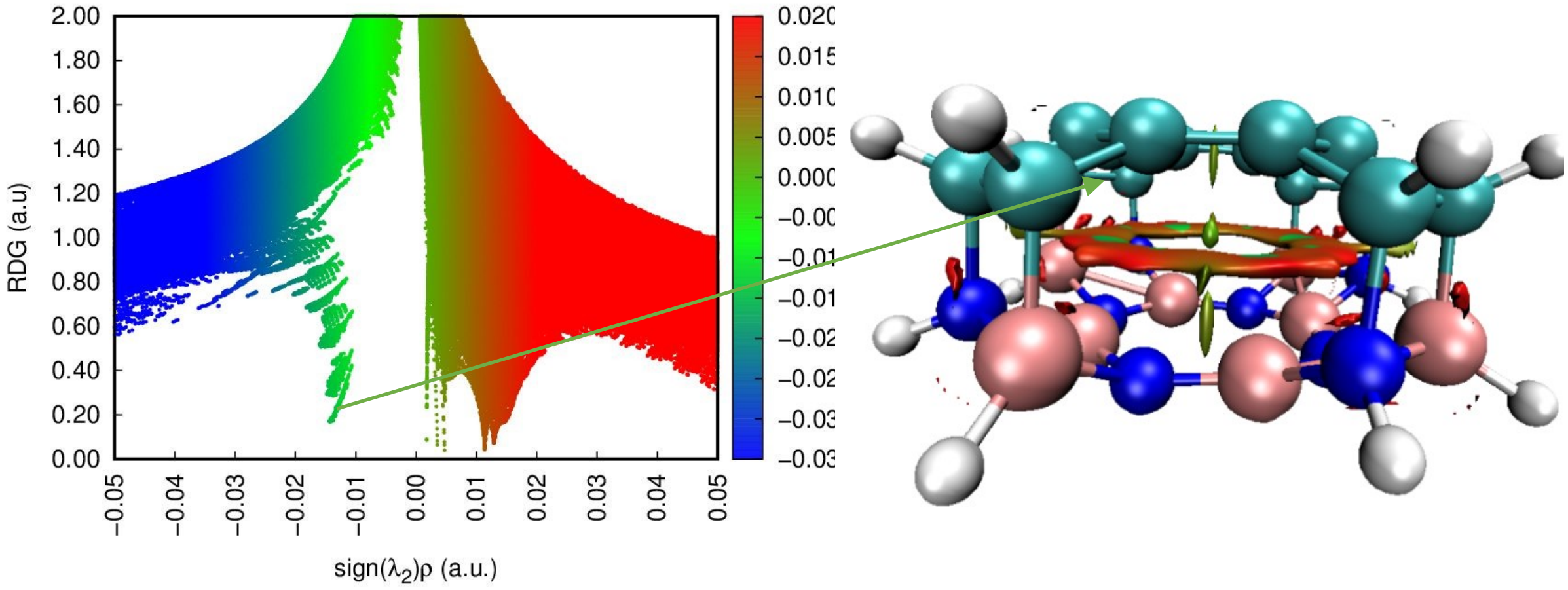

(d)

Figure 9: RDG scatter plot and isosurfaces of the complex structures (a) TG, (b) TBC_2N, (c) TBC_2N/TBN, (d) TG/TBN.

Supplementary Tables Using LANL2DZ Basis Set

Table 1: Bond lengths between C–C, C–B, C–N, and B–N atoms in tetragonal, octagonal, and shared structures of the MBL framework in two layers (L1 and L2).

Structure	Layer	Bond length									
		Tetragonal				Octagonal			Common bonds		
		C-C	C-B	C-N	B-N	C-C	B-N	C-C	C-B	C-N	B-N
TG	L1	1.55	…	…	…	1.46	…	1.38	…	…	…
	L2	1.55	…	…	…	1.46	…	1.38	…	…	…
TBC_2N	L1	…	1.59	1.49	…	1.36	1.41	…	1.54	1.45	…
	L2	…	1.59	1.49	…	1.36	1.41	…	1.54	1.45	…
TG/TBC_2N	L1	1.49	…	…	…	1.39	…	1.48	…	…	…
	L2	…	1.59	1.49	1.40	1.37	1.41	…	1.54	1.45	…
TG/TBN	L1	1.54	…	…	…	1.43	…	1.41	…	…	…
	L2	…	…	…	1.61	…	1.41	…	…	…	1.44

Table 2: Calculated molecular vibrational frequencies (cm^{-1}) following structural optimization.

**MBL Structures**	**Vibration Type**	**Frequencies of MBL structures** (cm^{-1})
TG	sp^2 C-C stretching	1569.45-1578.44
	sp^2 C-H stretching	(1259.54-3157.6) 1173.02-1259.54
	Inter-layer stretching	246.13(in/out) 336.13(L1-L2)
TBC_2N	sp^2 C-C stretching	(1739.48, 1650.41), 1625.80,1721.91
	B-C stretching	
	B-N stretching	1522.49, (1437.13-1508.51)
	B-H stretching	2728.96-2741.77
	N-H stretching	3520.21-3551.60
	sp^2 C-H stretching	3070.04-3112.04
	Inter-layer stretching	585.74
TG/TBC_2N	sp^2 C-C stretching	1517.05-1775.96
	B-N stretching	1465.14-1514.81
	B-H stretching	2718.95-2725.12
	N-H stretching	3542.64-3545.30
	sp^2 C-H stretching	3084.57-3254.17
	Inter-layer L1-L2 Stretching	608.01,
TG/TBN	sp^2 C-C stretching/ bending	1508.05-1612.24,
	B-N stretching	1299.75(1403.31), 1466.51
	B-H stretching	2598.46-2602.23
	N-H stretching	3566.61-3566.68

	sp^2 C-H stretching	3134.16-3157.19
	Inter-layer L1-L2 Stretching	285.69

Table 3: HOMO energy, LUMO energy, and energy gap of studied MBL structures.

Structure	HOMO energy (eV)	LUMO energy (eV)	Energy gap (eV)
TG	-4.574	-3.118	1.456
TBC_2N	-4.175	-3.852	0.323
TG/TBN	-4.542	-3.207	1.335
TG/TBC_2N	-4.410	-2.561	1.848

Table 4: Global Indices of the Optimized Geometries. Global hardness η_G, global softness β_G, chemical potential μ, the electrophilicity index of the structures ω.

MBL Structures	$Global\ hardness, \eta_G$ (eV)	$global\ softness, \beta_G$ (eV^{-1})	$Chemical\ potential, \mu$ (eV)	electrophilicity index of the structures, ω (eV)
TG	0.728	0.687	3.846	21.536
TBC_2N	0.162	3.086	4.0135	49.871
TG/TBC_2N	0.924	0.541	3.4855	22.450
TG/TBN	0.668	0.749	3.8745	20.040

Table 5: Electron density(ρ_e), $\nabla^2\rho_e$, electron's kinetic ED (G_e), potential ED (V_e), total ED(H_e), $-G_e/V_e$, and ratios in au unit at BC points of intermolecular bonds in the structures.

Structures	Bonds	ρ_e^{BC}	$\nabla^2\rho_e^{BC}$	G_e^{BC}	V_e^{BC}	H_e^{BC}	$-G_e^{BC}/V_e^{BC}$
TG	C_{27}-C_{28}	0.1898	-0.2675	0.0513	-0.1695	-0.1181	0.3029
	C_{14}-C_2	0.1898	-0.2675	0.0513	-0.1695	-0.1182	0.3027
	C_{25}-C_{26}	0.1898	-0.2674	0.0513	-0.1695	-0.1181	0.3028
	C_{15}-C_3	0.1898	-0.2676	0.0513	-0.1696	-0.1182	0.3027
	C_{20}-C_8	0.1898	-0.2676	0.0513	-0.1696	-0.1182	0.3027
	C_{33}-C_{34}	0.1898	-0.2674	0.0513	-0.1695	-0.1181	0.3028
	C_{17}-C_5	0.1898	-0.2675	0.0513	-0.1695	-0.1182	0.3027
	C_{37}-C_{38}	0.1898	-0.2675	0.0513	-0.1695	-0.1182	0.3027
T-BC_2N	C_5-C_{29}	0.1908	-0.2812	0.0494	-0.1691	-0.1197	0.2921
	C_3-C_{27}	0.1908	-0.2813	0.0494	-0.1691	-0.1197	0.2921
	C_{15}-C_{39}	0.1962	-0.2983	0.0513	-0.1772	-0.1259	0.2896
	C_9-C_{33}	0.1962	-0.2983	0.0513	-0.1773	-0.1259	0.2896
TG/T-BN	C_7-B_{39}	0.1395	-0.1595	0.0792	-0.1983	-0.1191	0.3994
	C_{14}-B_{40}	0.1396	-0.1592	0.0794	-0.1986	-0.1192	0.3998
	C_{23}-B_{38}	0.1396	-0.1592	0.0794	-0.1986	-0.1192	0.3998
	C_{12}-B_{37}	0.1396	-0.1592	0.0794	-0.1987	-0.1192	0.3998
	C_6-N_{48}	0.1872	-0.2125	0.0797	-0.2127	-0.1329	0.3750
	C_9-N_{44}	0.1873	-0.2127	0.0798	-0.2128	-0.1321	0.3750
	C_{17}-N_{47}	0.1875	-0.2135	0.0799	-0.2131	-0.1332	0.3748
	C_{21}-N_{41}	0.1875	-0.2135	0.0799	-0.2132	-0.1333	0.3748
TG/T-BC_2N	C_3-C_{26}	0.1995	-0.3186	0.0515	-0.1826	-0.1311	0.2819
	C_5-C_{27}	0.1995	-0.3186	0.0515	-0.1826	-0.1311	0.2819
	C_9-C_{29}	0.2016	-0.3270	0.0519	-0.1856	-0.1336	0.2797
	C_{15}-C_{32}	0.2016	-0.3270	0.0519	-0.1856	-0.1336	0.2797
